# Naphthalene Diimide–Tetraazacycloalkane Conjugates Are G-Quadruplex-Based HIV-1 Inhibitors with a Dual Mode of Action

**DOI:** 10.1021/acsinfecdis.3c00453

**Published:** 2024-01-04

**Authors:** Matteo Nadai, Filippo Doria, Ilaria Frasson, Rosalba Perrone, Valentina Pirota, Greta Bergamaschi, Mauro Freccero, Sara N. Richter

**Affiliations:** † Department of Molecular Medicine, University of Padua, Via Gabelli 63, 35121 Padua, Italy; ‡ Department of Chemistry, University of Pavia, V.le Taramelli 10, 27100 Pavia, Italy; § Buck Institute for Research on Aging, Novato, California 94945, United States; ∥ National Research Council of Italy, Istituto di Scienze e Tecnologie Chimiche “Giulio Natta” (SCITEC–CNR), Via Mario Bianco 9, 20131 Milano, Italy; ⊥ Microbiology and Virology Unit, Padua University Hospital, 35121 Padua, Italy

**Keywords:** naphthalene diimides, G-quadruplex, HIV, LTR, metal–organic complexes

## Abstract

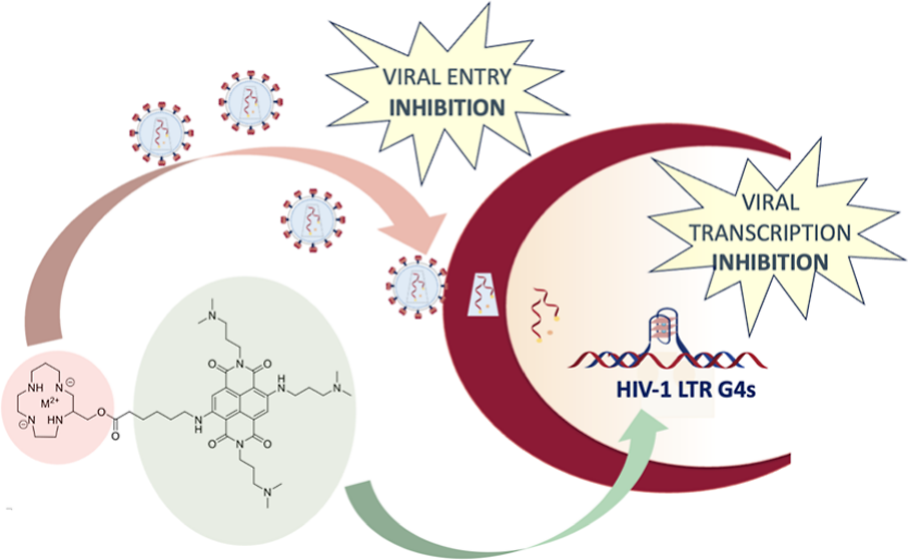

Human immunodeficiency virus 1 (HIV-1) therapeutic regimens consist of three or more drugs targeting different steps of the viral life cycle to limit the emergence of viral resistance. In line with the multitargeting strategy, here we conjugated a naphthalene diimide (NDI) moiety with a tetraazacycloalkane to obtain novel naphthalene diimide (NDI)–tetraazacycloalkane conjugates. The NDI inhibits the HIV-1 promoter activity by binding to LTR G-quadruplexes, and the tetraazacycloalkane mimics AMD3100, which blocks HIV entry into cells by interfering with the CXCR4 coreceptor. We synthesized, purified, and tested the metal-free NDI–tetraazacycloalkane conjugate and the two derived metal–organic complexes (MOCs) that incorporate Cu^2+^ and Zn^2+^. The NDI-MOCs showed enhanced binding to LTR G4s as assessed by FRET and CD assays in vitro. They also showed enhanced activity in cells where they dose-dependently reduced LTR promoter activity and inhibited viral entry only of the HIV-1 strain that exploited the CXCR4 coreceptor. The time of addition assay confirmed the dual targeting at the different HIV-1 steps. Our results indicate that the NDI-MOC conjugates can simultaneously inhibit viral entry, by targeting the CXCR4 coreceptor, and LTR promoter activity, by stabilizing the LTR G-quadruplexes. The approach of combining multiple targets in a single compound may streamline treatment regimens and improve the overall patient outcomes.

HIV-1, the causative agent of AIDS, is a retrovirus that integrates its RNA genome into the host DNA after reverse transcription.^1^ Cells that present on their surface the CD4 receptor and the alpha-chemokine CXCR4 or beta-chemokine CCR5 coreceptors, such as CD4+ T cells, are the natural target of the infection.^2^ Drugs that target several HIV-1 life cycle steps are now available.^3^ These drugs are very effective in inhibiting viral replication and thus help infected people live longer and healthier lives, but they are not a cure for HIV infection. In addition, resistance to each class of anti-HIV-1 drugs has been reported,^4,5^ and thus new drugs with new targets are highly sought.

G-quadruplexes (G4s) are a type of nucleic acid secondary structure that can form in G-rich sequences of DNA or RNA. They consist of four nucleic acid strands held together by hydrogen bonds between guanine bases, forming a square planar arrangement called a G-quartet. Multiple G-quartets can stack on top of each other to form a four-stranded helix with a central channel that can accommodate a cation, usually potassium. G-quadruplexes can adopt different topologies depending on the orientation and arrangement of the strands, as well as the length and composition of the loops that connect the tetrads.^6^ DNA G4s have been found in various genomic regions such as telomeres, gene promoters, and regulatory elements. They are involved in many biological processes, such as telomere maintenance, gene expression, DNA replication, recombination, and repair.^7^ They interact with various proteins that modulate their folding and function.^8^ G4s have also recently been reported in the genomes and transcripts of several viruses, including human immunodeficiency virus (HIV), hepatitis C virus (HCV), and SARS-CoV-2. These viral G-quadruplexes may play important roles in viral replication, transcription, translation, and packaging and hence hold promises in antiviral therapy.^9−12^

The HIV-1 genome contains several G-rich regions that can potentially form G4s, mainly in the long terminal repeat (LTR), the region that interacts with key transcription factors and controls transcription of all viral genes.^13−18^ DNA G4s in the LTR can act as silencer elements that inhibit viral transcription by blocking the binding of transcription factors or RNA polymerase.^13^ Their presence is highly regulated by the interaction with cellular proteins, such as nucleolin, hnRNP A2/B1, and FUS.^19−21^ HIV-1 RNA G4s can affect the viral genome structure and function:^14^ they are processed by the HIV-1 nucleocapsid protein NCp7, which remains associated with the viral RNA during reverse transcription and binds to and unfolds RNA G4s in the HIV-1 genome.^22,23^ G4s in HIV-1 represent a new frontier in virus pathogenesis and antiviral therapy. Targeting viral G4s with specific ligands could inhibit viral replication by disrupting key steps of the viral life cycle.

Several anti-HIV-1 G4 ligands have been identified and characterized. Some of these ligands have shown potent and selective inhibitory effects on HIV-1 promoter activity and replication.^14,24−29^ Anti-HIV-1 G4 ligands may thus offer a promising strategy to complement the current antiretroviral therapy and eradicate the latent viral reservoirs.

Naphthalene diimides (NDIs) are a class of G4 ligands that have shown high affinity and selectivity for G4 structures over duplex DNA.^30−33^ NDIs are planar aromatic molecules that can stack on the G-quartets of G4s and form π–π interactions.^34^ They also have one or two lateral chains that can form hydrogen bonds with the guanines and/or the loops of G4s.^35,36^ NDIs have unique optoelectronic properties, such as absorption and red-emission, that make them suitable for multimodal applications.^37^ Additionally, NDIs can be easily modified with functional groups (i.e., carboxylic acid or amine moieties) to achieve conjugation to cell penetrating peptides (CPP) and peptide nucleic acids (NDI–PNA conjugates),^29^ boosting their binding properties (affinity and selectivity) and cell entry.

Our group has recently exploited the conjugation of NDI with copper chelators to realize dual-mode agents for G4 targeting and cleavage.^38^ Based on our knowledge, we were inspired by the metal macrocycles properties as inhibitors of HIV-1 entry into the cells.^39^**AMD3100** (plerixafor), (1,1′-[1,4-phenylenebis(methylene)]-bis-1,4,8,11-tetraazacyclotetradecane octahydrochloride dehydrate), was found to prevent replication of T-tropic HIV at low nanomolar concentrations.^40^ This high potency is thought to be due to the polycationic nature of the bicyclam structure, which would favor the interaction with the predominantly negatively charged extracellular domain of CXCR4, and to the 1,4-phenylenebis(methylene) linker, which connects the two bicyclam rings.^41,42^ In addition, since the macrocyclic monomers bind strongly to Zn(II), it is believed that **AMD3100** exists as a Zn(II) complex in vivo.^43^**AMD3100** was the first small-molecule chemokine receptor antagonist approved for clinical studies.^44^

We hence envisioned engineering new metal tetraazacycloalkane–NDI conjugates to target both viral entry and LTR promoter activity to potently inhibit HIV infection.

## Results

### Rational Design and Synthesis

**Figure 1:**
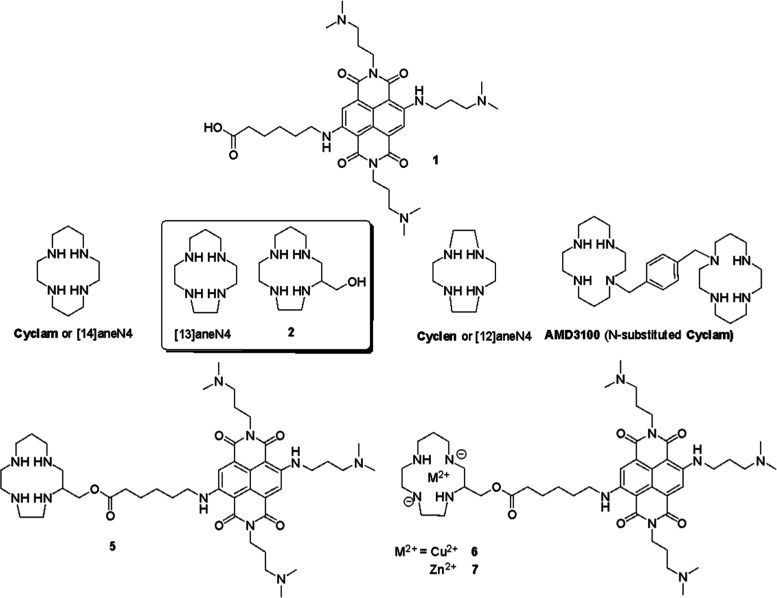
Structures of the naphthalene diimide **1** to be tethered to the tetraazacycloalkane **2**, yielding the metal-free NDI–tetraazacycloalkane conjugate **5** and the two NDI-MOCs incorporating Cu^2+^ (**6**) and Zn^2+^ (**7**). The structures of other tetraazacycloalkane Cu^2+^ ligands (**Cyclam**, **Cyclen,** and [13]aneN4) and of the drug **AMD3100** are shown for direct comparison with **2**.

**Scheme 1:**
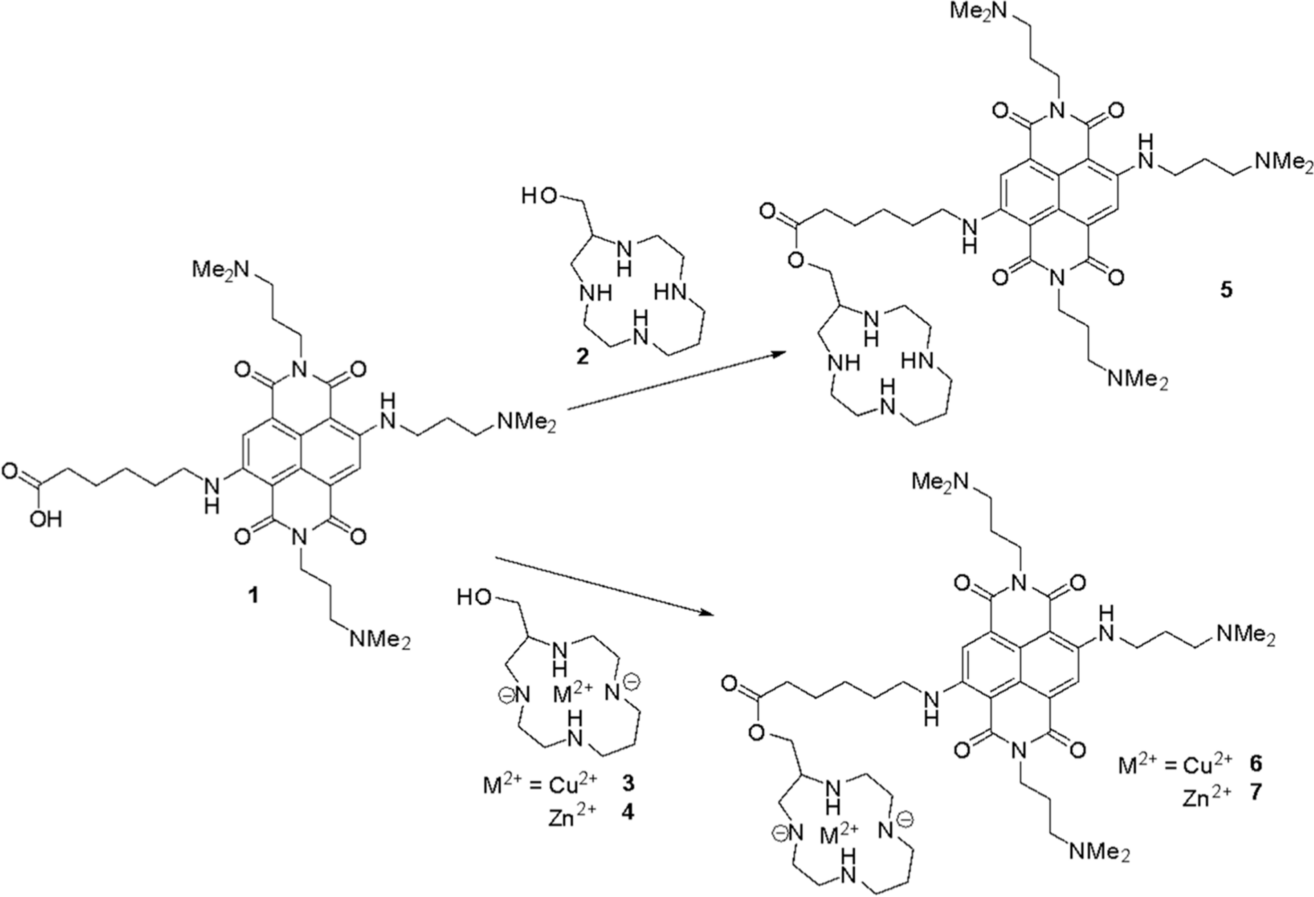
Synthesis of NDI–Tetraazacycloalkane Conjugates (**5–7**); Tetraazacycloalkane (**2–4**) as Starting Material, DMF, HATU, DIPEA, r.t., 3 h

We tethered NDI **1** to tetraazacycloalkane **2**, taking advantage of the flexible conjugation chemistry of NDIs embedding a carboxylic moiety. **2** is an hydroxymethyl analogue of [13]aneN4 (Figure 1), which stably embeds transition metal ions under physiological conditions, due to the cyclam-like binding cavity. The homologue **Cyclam** (Figure 1) and its derivatives block HIV entry into cells by binding specifically to the CXCR4 coreceptor. The most studied cyclam-derivative is **AMD3100**, a highly potent anti-HIV drug, containing two macrocyclic units linked with a xylyl spacer at the nitrogen atom. The antiviral activity of **AMD3100** correlates with the strength of the binding to the CXCR4 coreceptor and is enhanced by Zn^2+^ or Cu^2+^ metal complexation of the macrocycles^39^ or Ni^2+^.^49^**Cyclam** and **Cyclen** ([12]aneN4) are both well-known Cu^2+^ and Zn^2+^ ligands, with extremely high stability constants (log *K*_a_ 26.51 and 23.29, toward Cu^2+^, for **Cyclam** and **Cyclen,** respectively).^50,51^ Although the analogue [13]aneN4 derivative has been less widely used than its analogues **Cyclam** and **Cyclen**, it exhibits a very similar affinity for Cu^2+^ (log *K*_a_ 24.41).^51,52^ Unlike **Cyclam**, it allows for a straightforward functionalization using its derivative [13]aneN4-methanol (**2**, Figure 1). The latter exhibits a structural modification of the tetraazacycloalkane moiety at one C atom without significantly affecting the affinity for Cu^2+^. On the contrary, the most common functionalization of the **Cyclam** (i.e., **AMD3100**) is at the nitrogen atom, which is expected to remarkably reduce the log *K*_a_ toward Cu^2+^ (p*K*_a_ ∼ 19.7).^50^ Therefore, to preserve the high affinity of the tetraazacycloalkane for Cu^2+^, which is a key feature to avoid Cu^2+^ release in cell, we chose to conjugate the NDI **1** to **2**, as racemic mixture, according to the synthetic procedure outlined in Scheme 1, by condensation reaction of the NDI **1** to the tetraazamacrocyclic ligand **2**.

NDI **1** was prepared using an optimized six-steps protocol published by us.^29^ The 13aneN4 macrocycle functionalized at a C atom by a –CH_2_OH pendant arm (**2**), was achieved exploiting the bisaminal template approach successfully reported by Boschetti et al.^45^ and used as a racemate. To avoid side products, cation (Cu^2+^ or Zn^2+^) complexation by macrocycle **2** was obtained before the conjugation to NDI **1**. The metal organic complexes (MOCs) **3** and **4** were synthesized by refluxing **2** in the presence of a stoichiometric amount of metal triflate salt [Cu(CF_3_SO_3_)_2_ or Zn(CF_3_SO_3_)_2_] in *tert*-butanol for 1 h. Refluxing conditions are crucial for promoting the formation of the thermodynamically most stable species in transition metal plain complexes.^53^ The final conjugations between NDI **1** and macrocycles **2**, **3**, and **4** were performed with HATU as a condensing agent at room temperature for 3 h. The final products **5–7** obtained in good yields (>60%) were purified by reverse phase preparative HPLC. All compounds were >98.2% pure as assessed by HPLC analysis (see compounds’ characterization in Supporting Information).

### NDI–Tetraazacycloalkane Conjugates Specifically Stabilize HIV-1 LTR G-Quadruplexes

We have previously shown that the HIV-1 LTR promoter contains two G4 forming sequences, namely, LTR-III and LTR-IV, whose stabilization affects viral transcription.^13^ Both sequences are composed by 3 G-quartets:^15,54^ within the dynamic full length LTR sequence, LTR-III is the most stable G4 and LTR-IV folds in the presence of G4 ligands.^13^

We performed a FRET melting assay to assess the degree of stabilization of LTR G4s by the NDI-tetraazacycloalkane conjugates (**5–7**). The presence of a coordinated metal within the macrocyclic moiety boosted the compounds’ ability to stabilize viral G4s, as assessed by comparing both the Cu(II)- and Zn(II)-MOCs **6** and **7** with the metal free counterpart **5**. The most active compound was Cu(II)-MOC **6**, with the highest stabilization on the LTR G4s and the lowest on the telomeric G4 and dsDNA, the latter used as controls to address compound specificity toward the LTR G4 conformation (Table 1, Figure S1). Competition with increasing concentrations of dsDNA did not modify the binding of **5–7** toward LTR G4, confirming compounds specificity (Figure S2).

**Table 1:** FRET Melting Temperatures (*T*_m_, °C) of HIV-1 G4 Folding Sequences Measured by FRET in 100 mM KCl^a^

	*T*_m_ K^+^ 100 mM	*T*_m_ K^+^ 100 mM + **5**	Δ*T*_m_	*T*_m_ K^+^ 100 mM + **6**	Δ*T*_m_	*T*_m_ K^+^ 100 mM + **7**	Δ*T*_m_
LTR-III	65.2 ± 0.1	77.7 ± 0.1	12.5	86.3 ± 0.2	21.1	82.0 ± 0.1	16.8
LTR-IV	58.7 ± 0.5	73.9 ± 0.6	15.2	77.4 ± 0.5	18.7	70.9 ± 0.3	12.2
F21T	64.4 ± 0.4	66.6 ± 0.1	2.2	66.1 ± 0.2	1.7	65.1 ± 0.1	0.7
dsDNA	64.2 ± 0.1	65.0 ± 0.2	0.8	65.0 ± 0.1	0.8	65.0 ± 0.1	0.8

a Each sequence (0.25 μM) was analyzed in the absence and presence of the NDI–tetraazacycloalkane conjugates (**5–7**) (0.5 μM).

Circular dichroism analysis confirmed the high degree of G4 stabilization, with the Cu(II)-MOC **6** being more active in stabilizing the two viral G4-forming sequences, while slightly destabilizing the telomeric one (Table 2 and Figures S3–S6).

**Table 2:** CD Melting Temperatures (*T*_m_, °C) of HIV-1 G4 Folding Sequences Measured by Circular Dichroism in 100 mM KCl^a^

	*T*_m_ K^+^ 100 mM	*T*_m_ K^+^ 100 mM + **5**	Δ*T*_m_	*T*_m_ K^+^ 100 mM + **6**	Δ*T*_m_	*T*_m_ K^+^ 100 mM + **7**	Δ*T*_m_
LTR-III	68.2 ± 0.1	78.6 ± 0.5	10.4	81.7 ± 0.5	13.5	75.5 ± 0.6	7.3
LTR-IV	50.4 ± 0.4	71.6 ± 0.4	21.2	>90	>39.6	62.9 ± 0.3	12.5
Tel22	66.8 ± 0.2	60.9 ± 0.3	–5.9	63.5 ± 0.2	–3.3	66.2 ± 0.4	–0.6

a Each sequence (4 μM) was analyzed in the absence and presence of the NDI–tetraazacycloalkane conjugates (**5–7**) (8 μM).

The Cu(II)-MOC **6** selectivity toward the two viral G4-forming sequences might be ascribed to the presence of Cu(II), which offers an additional coordination site for a conformational free nucleobase in close proximity. The stem loop, which is a key feature of G4 LTR-III^54^ and probably the 5-base long lateral loop of the G4 LTR-IV,^15^ may both offer the required nucleobase conformational freedom for Cu(II)-binding. This additional binding mode hypothesis is suggested by our previous evidence on a similar NDI (**NDI-Cu-DETA**), exhibiting an embedded Cu(II) binding moiety. Indeed, **NDI-Cu-DETA** selectively binds (1:1 stoichiometry) and cleaves G4 LTR-III in close proximity to the duplex-quadruplex junction, where the nucleobases exhibit great conformational mobility.^38^

### Inhibition of LTR Promoter Activity by the NDI–Tetraazacycloalkane Conjugates

**Figure 2:**
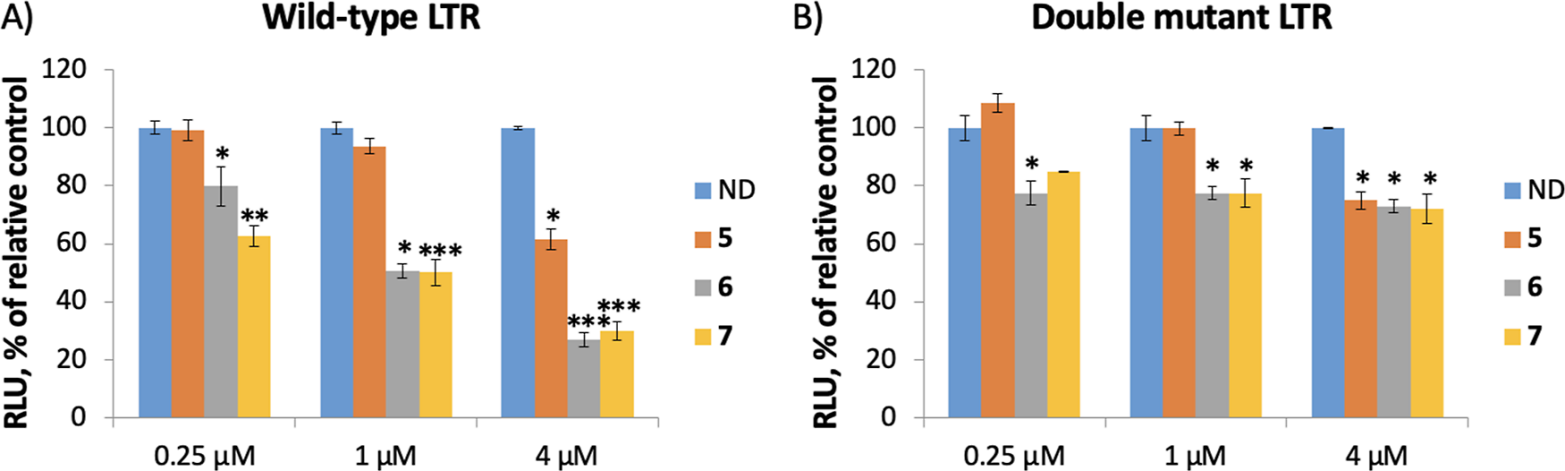
Effects of NDI–tetraazacycloalkane conjugates **5–7** on the LTR promoter activity. (A,B) Luciferase assay on HEK 293T cells transiently transfected with plasmids containing the wild-type (A) or double mutant (B) LTR promoter sequence upstream a luciferase reporter gene. Luciferase signals, expressed in relative light units (RLU), were measured as a function of the increasing concentrations of **5–7** and normalized to the total protein content. Histograms represent the mean of three independent biological replicates, each in duplicate. ND = no drug; * = *p* value < 0.05; ** = *p* value < 0.01; *** = *p* value < 0.001.

We next tested the effect of the NDI–tetraazacycloalkane conjugates on the LTR promoter activity in cells. The wild-type (wt) LTR that folds into G4 and a double mutant LTR unable to fold into G4 were inserted in luciferase reporter plasmids,^13^ which were transiently transfected into HEK 293T cells either alone or in the presence of increasing amounts of **5**, **6,** and **7**. Analysis of the luciferase signal showed high dose-dependent inhibition on wt LTR promoter activity by the metal-coordinated compounds (up to ∼25% of the untreated control), while a lower effect on the double mutant LTR (Figure 2). These results indicate that the NDI-MOCs **6** and **7** and, to a lower extent, the metal free NDI–tetraazacycloalkane conjugate **5**, inhibit LTR promoter activity by interacting with the LTR G4s.

### NDI–Tetraazacycloalkane Conjugates Display Potent Antiviral Activity

Encouraged by these results, we tested the compounds’ activity in infected cells. We used TZM-bl cells that are HeLa cells stably expressing large amounts of CD4, CXCR4, and CCR5, thus being highly susceptible to HIV-infection, with integrated luciferase and beta-galactosidase genes under the control of the HIV-1 promoter. We first tested NDI–tetraazacycloalkane conjugates’ cytotoxicity on TZM-bl cells by treating them with increasing amounts of compounds (0.097–25 μM) for 24 and 48 h. Cytotoxicity curves were similar between the compounds with and without coordinated metal (Figure S7), indicating that the presence of the metal does not interfere with cell viability.

We next tested the antiviral activity of the NDI–tetraazacycloalkane conjugates in TZM-bl cells. Cells were infected with HIV-1 NL4–3 or BaL, two strains with different cell tropism due to the different coreceptors that are bound by the virus to get access into the cell: HIV-1 NL4–3 exploits the CXCR4 coreceptor (X4 strain), while HIV-1 BaL exploits the CCR5 coreceptor (R5 strain). Compounds were administered at the time of infection or 2 h post infection, and the antiviral activity was evaluated 24 and 48 h post infection. Cytotoxicity was tested in parallel on uninfected cells to assess compound cytotoxicity.

As shown in Table 3 and Figures S8 and S9, the NDI–tetraazacycloalkane conjugates were more active toward the HIV-1 X4 strain than the R5 strain: on the HIV-1 X4 strain, Cu(II)-MOC **6** had the greatest antiviral activity, with IC_50_ in the nanomolar range, followed by Zn(II)-MOC **7**. Both compounds were about twice as active when administered at the time of viral entry into the cell (0 hpi) compared to 2 h later. The metal-free NDI–tetraazacycloalkane conjugate **5** was about 12 times less potent in the 24 h incubation assay and 5 times less potent in the 48 h incubation assay. On the HIV-1 R5 strain, the NDI-macrocycle compounds displayed lower antiviral activity, with IC_50_ values always above 10 μM. Their potency on this HIV-1 strain was comparable, with Zn(II)-MOC **7** being slightly more active. None of the compounds displayed cytotoxicity on the mock-infected TZM-bl cells upon 24- and 48 h incubation (CC_50_ > 25 μM). These data reinforce the observation that the presence of the metals is necessary for the antiviral activity.

**Table 3:** Antiviral Assay on HIV-1 Infected Cells^a^

compound	virus	strain	hpi (administration)	hpi (evaluation)	IC_50_ (μM)	CC_50_ (μM)	SI
**5**	HIV-1	X4	0	24	17.76 ± 0.94	>25	>1.41
	HIV-1	X4	2	24	12.1 ± 1.02	>25	>2.07
**6**	HIV-1	X4	0	24	0.56 ± 0.17	>25	>44.9
	HIV-1	X4	2	24	0.68 ± 0.21	>25	>36.82
**7**	HIV-1	X4	0	24	0.57 ± 0.12	>25	>43.40
	HIV-1	X4	2	24	0.95 ± 0.09	>25	>26.31
**5**	HIV-1	R5	0	24	>25	>25	na
	HIV-1	R5	2	24	>25	>25	na
**6**	HIV-1	R5	0	24	>25	>25	na
	HIV-1	R5	2	24	20.75 ± 2.19	>25	>1.20
**7**	HIV-1	R5	0	24	>25	>25	na
	HIV-1	R5	2	24	10.70 ± 1.55	>25	>2.34
**5**	HIV-1	X4	0	48	1.86 ± 0.49	>25	>13.44
	HIV-1	X4	2	48	1.82 ± 0.65	>25	>13.71
**6**	HIV-1	X4	0	48	0.35 ± 0.04	>25	>72.11
	HIV-1	X4	2	48	0.69 ± 0.13	>25	>36.06
**7**	HIV-1	X4	0	48	0.42 ± 0.06	>25	>59.52
	HIV-1	X4	2	48	0.94 ± 0.16	>25	>26.50
**5**	HIV-1	R5	0	48	15.11 ± 1.21	>25	>1.65
	HIV-1	R5	2	48	16.60 ± 1.33	>25	>1.51
**6**	HIV-1	R5	0	48	16.36 ± 0.97	>25	>1.53
	HIV-1	R5	2	48	>25	>25	na
**7**	HIV-1	R5	0	48	11.00 ± 1.04	>25	>2.27
	HIV-1	R5	2	48	11.03 ± 1.10	>25	>2.26

a TZM-bl cells were infected with the HIV-1 X4 strain (NL4-3) or R5 strain (BaL), at MOI values of 0.5 and 0.3, respectively. Compounds were administered at the time of infection or 2 h post infection, and the antiviral activity was evaluated 24 and 48 h post infection. An MTT assay was performed in parallel on uninfected cells to assess compound cytotoxicity. IC_50_ is the concentration of test compound that inhibits 50% of LTR-luciferase signal, CC_50_ is the concentration of test compound that reduces the absorbance of the mock-infected cells by 50%. hpi means hours post infection. SI is the selectivity index (CC_50_/IC_50_ ratio). na stands for not applicable.

Controls including **AMD3100**, a tetraazacycloalkane mimic, and the individual parental inhibitors NDI were also performed. AMD3100 displays excellent activity against the pNL4.3 strain, while it is inactive against the BaL strain (Figure S10). On the other hand, the parental NDI was highly cytotoxic, hence it could not be evaluated further for its antiviral activity (Figure S11). These data indicate that the conjugation of the NDI with the **AMD3100** moiety, besides making the new molecule a double-edge sword, also highly decreases its cytotoxicity.

We also excluded that the observed antiviral activity depended on the presence of the metal by assessing both viral replication and cytotoxicity upon treatment with a wide range of CuSO_4_ concentrations (Figure S12).

### NDI-MOCs **6** and **7** Target Both the Entry and Replication Steps of HIV-1 X4 Strain

The antiviral assays highlighted that NDI-MOCs **6** and **7** were the most potent compounds against the HIV-1 X4 strain, while all compounds were much less active against the R5 strain. To pinpoint the viral steps targeted by NDI-MOCs **6** and **7** during infection, we set up a time of addition assay, where compounds are administered to infected cells at different times post infection up to the time when a single round of HIV-1 replication is completed.^55^ This assay indicates how long the addition of a compound can be postponed before it loses its antiviral activity: comparing the test compounds with reference drugs, whose mechanism is well characterized in the time frame of virus replication, it is possible to determine which viral steps are affected by the test compounds.^56^

**Figure 3:**
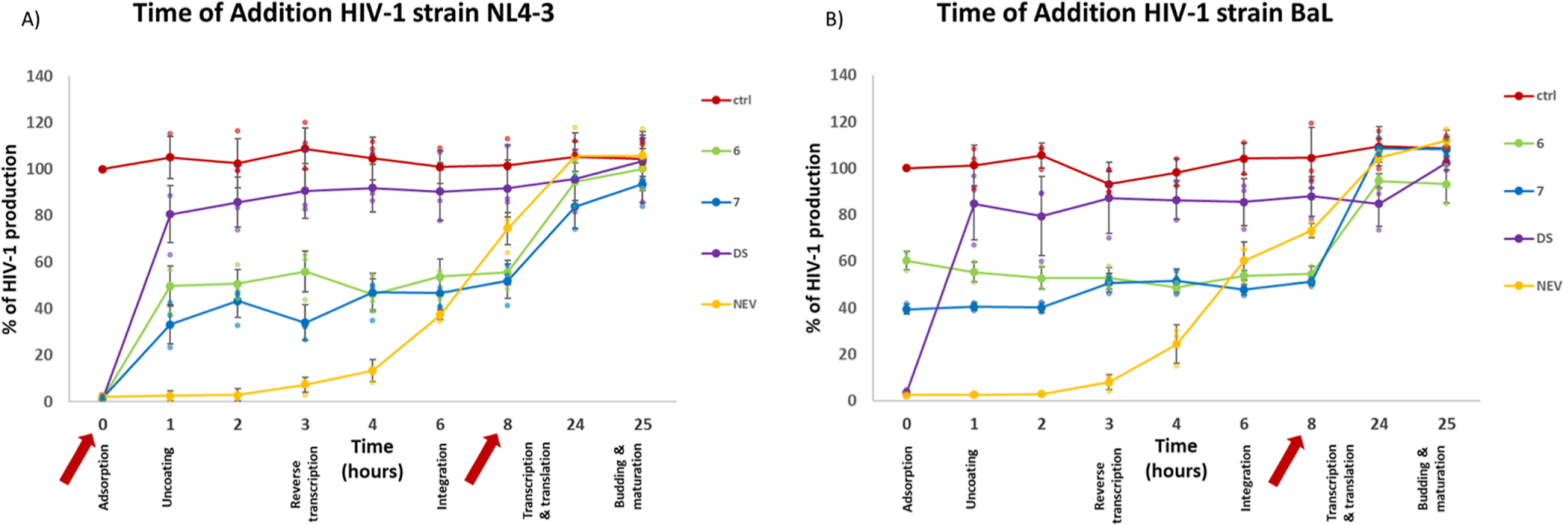
Time of addition (TOA) assay. TZM-bl cells were infected with the HIV-1 X4 strain (NL4–3) (A) or the R5 strain (BaL) (B) and NDI-MOCs **6** and **7** were added at different time points after infection. 25 h post infection HIV-1 production was assessed following the LTR-luciferase signal using the britelite plus Reporter Gene Assay System. The activity of the test compounds was compared with that of untreated infected cells and reference drugs (DS stands for dextran sulfate and NEV stands for nevirapine). These data are representative of three independent biological replicates.

TZM-bl cells were infected with both HIV-1 X4 and R5 strains, and NDI-MOCs were administered every hour, as shown in Figure 3. Virus production was monitored at 25 h post infection, i.e., at the end of a single round of HIV-1 replication, by exploiting the LTR-driven luciferase reporter system. On the HIV-1 X4 strain (Figure 3A), NDI-MOCs **6** and **7** were active both when added at the time of infection (time 0) and after 8 h. The virus production curve showed a first jump between 0 and 1 h post infection, indicating that the entry step is targeted, and then a second jump after 8 h post infection, indicating inhibition of HIV-1 in the steps occurring up to 8 h post infection. These data indicate that both virus entry and postintegration steps are affected. Since the LTR promoter is active up to about 8 h post infection, our results agree with the previous spectroscopic and luciferase data, indicating a G4-mediated effect on HIV-1 LTR. In contrast, on the HIV-1 R5 strain, the NDI-MOCs had no effect on the entry step (Figure 3B), but they retained activity up to 8 h post infection, as indicated by the jump in the virus production curve between 8 and 24 hpi. These data confirm the ability of NDI-MOCs **6** and **7** to target postintegration steps also in this strain. Treatment with the control drugs confirmed that the entry inhibitor dextran sulfate was active at the time of infection only, while the retrotranscriptase inhibitor nevirapine maintained its activity up to 3–4 h post infection.

Overall, our results indicate that the NDI-MOCs can simultaneously target two HIV-1 steps, thus acting both as inhibitors of the CXCR4 coreceptor and as inhibitors of LTR activity by binding to LTR G4s.

## Discussion and Conclusions

The quest for an effective cure for HIV/AIDS has been ongoing for decades. Over the years, significant progress has been made in the development of antiretroviral therapies (ART) that target specific stages of the HIV life cycle. However, the emergence of drug resistance and the persistence of viral reservoirs have challenged the efficacy of single-target therapies. In response to these challenges, the administration of multiple drugs with different targets at different stages of the viral life cycle has been introduced and shown to work well and avoid or largely postpone the emergence of resistance.^57^ One possible alternative to the administration of multiple drugs would be the administration of a single drug with multiple targets. It has been shown that dual-target compounds exhibit superior antiviral activity compared to single-target drugs.^58,59^ In this line of antiviral drug development, we have designed and synthesized an NDI–tetraazacycloalkane conjugate (**5**), which tethers an NDI moiety that binds to HIV-1 LTR G4s, to the [13]aneN4 moiety that mimics **AMD3100**, a cyclam-containing macrocyclic antiviral drug that blocks HIV entry into cells by binding the CXCR4 alpha-chemokine coreceptor.^39^ The conjugation of the cyclam analogue to NDI has been synthetically obtained to retain its ability to stabilize the most relevant G4 structures (LTR-III and LTR-IV) present in the HIV-1 LTR promoter. The chosen tetraazacycloalkane [13]aneN4 moiety within the (NDI)–tetraazacycloalkane derivative (**5**) was capable of binding Cu^2+^ and Zn^2+^ with high affinity, a feature that allowed us to isolate and purify the resulting NDI-MOCs **6** and **7**, respectively. The high metal affinity is an extremely favorable feature that ensures that no free metal is released into the cell. Both NDI-MOCs **6** and **7** retained LTR G4 stabilization properties paralleled by excellent selectivity in comparison to the telomeric G4.

The improved LTR G4 stabilization due to the addition of the metal to the cyclam ring agrees with literature data, showing that the coordination of Cu^2+^ to the metal-binding sites of a tetradentate macrocycle increases the DNA affinities by more than 1 order of magnitude. It was speculated that this may be due to covalent metal–DNA binding. There are also many examples for Cu^2+^ complexes with a square-pyramidal coordination geometry due to an axial water molecule or counterion that can interact with DNA via intercalation or groove binding, often supported by additional H bonding interactions.^60^ The binding of metal complexes to G4-DNA has been extensively studied by the Vilar group,^61^ which reported that Pt(II) complexes effectively bind to G4 DNAs due to their square planar structure.

Both of our NDI-MOCs were the most potent compounds not only in vitro but also in cells. The compounds were shown to act at the LTR promoter in cells, as they inhibited the LTR promoter activity in transiently transfected cells. This activity depended on the G4s, as the double mutant unable to form LTR-III and LTR-IV G4s was much less affected. However, due to the richness in G bases of the LTR sequence, even upon the double mutations, other less stable G4s might form (with G-tracts formed by two instead of three Gs), which could explain the residual activity observed on the double mutant.

In infected cells, the compounds were shown to inhibit much more efficiently the HIV-1 strain that exploits the cell alpha-chemokine coreceptor CXCR4, which is targeted by the cyclam-derivative **AMD3100**. At the same time, no cytotoxicity was observed, supporting compounds selectivity. On the HIV-1 X4 strain, the NDI-MOCs were up to five times more potent that the metal-free NDI–tetraazacycloalkane conjugate **5**, indicating that complexation with the metal is key to the enhanced antiviral activity at the entry step. This is also attested by the lack of differential activity of **5** when administered at 0 and 2 hpi. Interestingly, conjugation of AMD3100 to the NDI mojety is also critical to decrease cytotoxicity: in fact, the parental NDI was too cytotoxic to warrant antiviral studies.

The time of addition (TOA) assay confirmed the double mechanism of action: the NDI-MOCs were active only against the strain that exploits the CXCR4 coreceptor (X4 strain) and up to 8 h post infection, which is the postintegration time when the LTR promoter is active, as gathered by comparison with the known drug nevirapine.^14,28^ This assay also indicates that the RNA LTR G4s, which would affect retrotranscription activity if stabilized by G4-ligands,^14,22^ were likely not involved in the mechanism of action of the NDI-MOCs, as we did not record an enhanced effect at the time when retrotranscription takes place (around 2–6 h), attested by the control antiretrotranscriptase drug nevirapine.

We also observed that targeting of the LTR G4s inhibited HIV-1 replication by about 50–70%, as shown in the 1–8 h time range of postponed treatment, while when cell entry was also targeted, i.e., in the HIV-1 NL4–3 strain at 0 h, inhibition was complete (100%). These data prove that the double targeting embedded in a single compound enhances the overall antiviral effects.

To our knowledge, this is the first evidence of compounds acting as HIV-1 antivirals through a dual mode of action, inhibiting simultaneously (i) the viral entry, targeting the CXCR4 coreceptor, and (ii) the LTR activity by LTR G-quadruplex stabilization.

The approach of combining multiple targets in a single compound can streamline treatment regimens and potentially enhance antiviral activity, delay drug resistance, suppress viral reservoirs, and improve adherence to the therapy and overall patient outcomes.

## Materials and Methods

### Materials and General Procedures

Reagents, solvents, and chemicals were purchased from Alfa Aesar or Sigma-Aldrich and were used as supplied without further purification.

TLC analysis was carried out on silica gel (Merck 60F-254) with visualization at 254 and 366 nm. HPLC analysis was performed using an Agilent SERIES 1260 system SERIES 1260. The column was XSelectHSS C18 (2.5 μm) (50 mm × 4.6 mm) (Waters). Flow was 1.4 mL/min. The following method was used (method 1): aqueous solvent: 0.1% trifluoroacetic acid in water; organic solvent: acetonitrile; gradient: 95% aqueous, gradually to 40% aqueous over 8 min and then isocratic flow for 4 min. NDI **1** was purified by semipreparative HPLC, specifically a Waters system combining a Delta 600 PUMP, a 2489 UV/vis detector, and a Fraction Collector III. An XSelect CSH Prep Phenyl-Hexyl 5 μm (150 × 30 mm) column (Waters) was used, with a flow of 27 mL/min. Purification was performed through the following method (method 2): aqueous solvent: 0.1% trifluoroacetic acid in water; organic solvent: acetonitrile; gradient: 95% aqueous, gradually to 92% aqueous over 4 min, then gradually to 70% aqueous over 14 min. NDI-macrocycle **6** and **7** conjugates were purified by preparative HPLC, specifically an Agilent system combining a Delta 600 PUMP, a 2489 UV/vis detector, and a Fraction Collector III. A Sunfire C18 (150 mm × 30 mm) column was used with a 30 mL/min flow. Purification was performed through the following method (method 3): aqueous solvent: 0.1% trifluoroacetic acid in water; organic solvent: acetonitrile; gradient: 95% aqueous, gradually to 50% aqueous over 16 min, then gradually to 0% aqueous over 5 min, and at the end isocratic flow for 1 min ^1^H-, ^13^C NMR spectra were recorded on a Bruker ADVANCE NEO instrument at 700 MHz. Mass spectrometry measurements were performed on a Thermo LTQ-XL (linear ion trap mass detector) with an electrospray ionization (ESI) source.

### Synthesis of [M^2+^-Macrocycle]CF_3_SO_3_ Complexes **3** and **4**

The (1,4,7,10-tetraazacyclotridec-5-yl)-methanol macrocycle was synthesized according to a published procedure^45^ and macrocycle complexation was obtained by a slight modification of the procedure reported in the literature.^46^ To a solution of the macrocycle (0.03 mmol) in 10 mL of *tert*-butanol, 1 equiv of M^2+^(CF_3_SO_3_)_2_ (0.03 mmol, M^2+^ = Cu^2+^ or Zn^2+^) was added as a solid. The mixture was heated at reflux for 1 h. Then the solvent was concentrated, and the addition of an excess of Et_2_O induced precipitation of the pure complex, which was collected by filtration.

### Synthesis of the NDI–Tetraazacycloalkane Conjugates

The precursor NDI **1** used was synthesized according to a published procedure.^29^ NDI **1** (0.03 mmol) was dissolved in 2 mL of DMF, by adding 0.035 mmol of HATU and 0.3 mmol of DIPEA. The mixture was stirred at room temperature for 20 min to ensure the carboxylic group activation, and then 0.033 mmol of the relative macrocycle was included. The mixture was stirred for 3 h, and the reaction progress was checked by analytical HPLC, using method 1. Once the reaction is complete, the mixture was diluted in acidic water (0.1% TFA) and purified by preparative HPLC, using method 2 for the NDI–tetraazacycloalkane conjugate **5** and 3r for its NDI-analogues **6** and **7**.

#### NDI–Tetraazacycloalkane Conjugate **5**

Yield: 51%. Blue solid. HPLC purity: 98.3%, rt = 4.6 min ^1^H NMR (700 MHz, CD_3_OD): δ 8.13 (s, 2H), 4.31–4.28 (m, 4H), 4.18–4.07 (m, 2H), 3.83 (quin, 1H), 3.69 (t, 2H), 3.59–3.56 (m, 4H), 3.39–3.34 (m, 10H), 3.29–3.21 (m, 8H); 2.93 (s, 6H); 2.91 (s, 6H); 2.89 (s, 6H); 2.37 (t, 2H), 3.32 (t, 2H); 2.26–2.17 (m, 6H), 1.84 (quin, 2H), 1.73 (quin, 2H), 1.64 (quin, 2H), 1.61–1.58 (m, 2H). ^13^C NMR (75 MHz, CD_3_OD): δ 174.1; 165.9; 163.2; 161.7; 161.5; 149.2; 148.6; 125.6; 121.3; 118.2; 117.7; 117.3; 116.0; 102.1; 101.3; 69.7; 65.0; 62.6; 55.5; 55.4; 55.3; 48.1; 48.2; 47.8; 42.4; 42.3; 42.2; 42.0; 39.6; 36.8; 36.7; 33.4; 31.7; 29.1; 28.5; 26.4; 24.8:24.7; 24.6; 23.4; 23.6.

#### NDI–Tetraazacycloalkane Conjugate MOC **6** (M = Cu^2+^)

Yield: 75%. Blue solid. HPLC purity: 100%, rt = 4.9 min. Mass characterization, *m*/*z* found (calcd): 1038.5 (1038.69) [**6** + CF_3_COOH]^+^, 970.42 (970.66) [**6** + 2Na]^+^, 960.67 (960.71) [**6** + 2H_2_O]^+^, 863.83 (863.14) [demetalled-**6**]^+^, 519.83 (519.84) [**6** + CF_3_COOH]^2+^, 485.75 (485.33) [**6** + 2Na]^2+^, 480.92 (480.85) [**6** + 2H_2_O]^+^, 462.92 (462.84) [**6**]^2+^, 432.5 (432.07) [demetalled-**6**]^2+^, 324.17 (324.22) [**6** + 2Na]^3+^, 308.92 (309.56) [**6**]^3+^, 231.92 (232.42) [**6**]^4+^.

#### NDI–Tetraazacycloalkane Conjugate MOC **7** (M = Zn^2+^)

Yield: 73%. Blue solid. HPLC purity: 98.7%, rt = 4.87 min. Mass characterization, *m*/*z* found (calcd): 999.25 (999.57) [**7** + 4H_2_O]^+^, 971.75 (971.48) [**7** + 2Na]^+^, 961.67 (961.96) [**7** + 2H_2_O]^+^, 486.42 (486.24) [**7** + 2Na]^2+^, 481.33 (481.48) [**7** + 2H_2_O]^2+^, 324.58 (324.49) [**7** + 2Na]^3+^, 243.67 (243.62) [**7** + 2Na]^4+^, 232.17 (231.88) [**7**]^4+^.

### FRET Analysis

FRET assay was performed with FAM (6-carboxyfluorescein) 5′-end- and Tamra (6-carboxytetramethylrhodamine) 3′-end-labeled oligonucleotides (Table S1). Fluorescence melting curves were determined with a LightCycler II (Roche) real-time PCR machine, using a total reaction volume of 20 μL, with 0.25 μM tagged oligonucleotide in a buffer containing 10 mM lithium cacodylate pH 7.4 with 100 mM KCl, in the presence or absence of 0.5 μM NDI–tetraazacycloalkane conjugate. After a first equilibration step at 30 °C during 2 min, a stepwise increase of 1 °C every minute for 65 cycles to reach 95 °C was performed and measurements were made after each cycle with excitation at 470 nm and detection at 530 nm. Final analysis of the data was carried out using Excel and Sigma Plot software. Oligonucleotides melting was monitored by observing emission of FAM, which was normalized between 0 and 1: *T*_m_ was defined as the temperature at which the normalized emission is 0.5. *T*_m_ values were mean of 2–3 experiments and Δ*T*_m_ was calculated as the difference *T*_m_ in the presence and absence of the compound.

### Circular Dichroism

All oligonucleotides used in this study were from Sigma-Aldrich (Milan, Italy) (Table S2). For CD analysis, oligonucleotides were diluted to a final concentration of 4 μM in lithium cacodylate buffer (10 mM, pH 7.4) and KCl 100 mM. After the annealing step (95 °C for 5 min), DNA samples were gradually cooled to room temperature and compounds added from stock at final concentration of 8 μM. CD spectra were recorded on a Chirascan-Plus (Applied Photophysics, Leatherhead, UK) equipped with a Peltier temperature controller using a quartz cell of 5 mm optical path length and an instrument scanning speed of 50 nm/min over a wavelength range of 230–320 nm. The reported spectrum of each sample represents the average of 2 scans and is baseline-corrected for signal contributions due to the buffer. Observed ellipticities were converted to mean residue ellipticity (θ) = deg × cm^2^ × dmol^–1^ (mol ellip). For the determination of *T*_m_, spectra were recorded over a temperature range of 20–90 °C, with a temperature increase of 5 °C/min. *T*_m_ values were calculated according to the van’t Hoff equation, applied for a two-state transition from a folded to unfolded state, assuming that the heat capacity of the folded and unfolded states are equal.

### Virus Stocks

HIV-1 NL4–3 and HIV-1 BaL stocks were prepared by transfection of HEK 293T with proviral genomes (obtained through the NIH AIDS Reagent Program, Division of AIDS, NIAID, NIH: HIV-1 NL4–3 Infectious Molecular Clone (pNL4–3) from Dr. Malcolm Martin (Cat# 114))^47^ (obtained through the NIH AIDS Reagent Program, Division of AIDS, NIAID, NIH).^48^

### Luciferase Assay

Luciferase assay was performed on transiently transfected human embryonic kidney 293T (HEK293T) cells. The HIV-1 LTR region (wt or M4 + 5) was inserted into the promoterless luciferase reporter vector pGL4.10-Luc2 (Promega Italia, Italy), as previously reported.^13^ For assessment of luciferase activity, HEK293T cells were seeded in a 12-well plate (2 × 10^5^ cells/well). Then 24 h later, cells were transfected with 120 ng/well of pGL4.10-LTRwt or of pGL4.10-LTR-M4 + 5 using TransIT-293 transfection reagent (Mirus Bio LLC, Madison, WI, USA), according to manufacturer’s protocol. After 1 h, cells were treated with compounds for 24 h at various concentrations (0.25–4 μM). Luciferase activity was measured using the britelite plus Reporter Gene Assay System (PerkinElmer Inc., Milan, Italy) at a Victor X2 multilabel plate reader (PerkinElmer Inc., Milan, Italy), according to manufacturer’s instructions. Cells were lysed in RIPA buffer (50 mM Tris–HCl pH 7.2, 150 mM NaCl, 1% Igepal, 0.1% SDS) and protein concentration was determined by a BCA assay (Thermo Scientific Pierce, Monza, Italy). Luciferase signals were subsequently normalized to total protein content, according to the manufacturer’s protocol.

### Cytotoxicity Assay

Cytotoxicity of tested compounds was performed in parallel to antiviral and reporter assays by MTT. The 50% cytotoxic concentration (CC_50_) was defined as the concentration of the test compound that was able to reduce the absorbance of the mock-infected/untreated cells by 50%. The 50% inhibitory concentration (IC_50_) was defined as the concentration of the test compound that inhibits 50% of the LTR-luciferase signal. The selectivity index (SI) is the relative effectiveness of the tested compound in inhibiting viral replication compared to inducing cell death (CC_50_ value/IC_50_ value). The therapeutic window (TW) is the concentration range at which the compound shows antiviral activity paralleled by less than 80% cytotoxicity. IC_50_ and CC_50_ values were calculated by using a nonlinear regression model.

### Antiviral Assay in HIV-1 Infected TZM-bl Cells

HIV-1 infectivity was measured using the TZM-bl reporter cell line (obtained through the NIH AIDS Reagent Program, Division of AIDS, NIAID, NIH: TZM-bl cells (catalog no. 8129) from Dr. John C. Kappes, and Dr. Xiaoyun Wu). TZM-bl is a HeLa cell line stably expressing large amounts of CD4 and CCR5, constitutively expressing CXCR4 and containing integrated copies of the luciferase and β-galactosidase genes under the control of the HIV-1 promoter. TZM-bl were grown in DMEM supplemented with 10% FBS. Cells (1 × 10^4^ cells/well) were seeded in 96-well plates and grown overnight to permit adherence prior to treatment and viral infection. Cells were next infected with HIV-1 NL4–3 strain or BaL strain at a MOI of respectively 0.5 and 0.3 for 1 h at 37 °C, treated with serial dilutions of tested compounds, and incubated at 37 °C. MOI values had been optimized in previous in-house experiments in order to obtain a well detectable virus release but avoid a virus-induced cytopathic effect. Compounds were reconstituted in DMSO, and dilutions were made in cell medium to maintain the same percentage of DMSO in all dilutions. Untreated cells were treated with the same solvent. After 24 or 48 h, cells were washed with PBS 1× and HIV-1 production was assessed following the LTR-luciferase signal using the britelite plus Reporter Gene Assay System (PerkinElmer, Waltman, MA, USA) according to the manufacturer’s protocol.

### TOA Experiments

TZM-bl cells (1 × 10^4^ cells/well) were seeded in 96-well plates and grown overnight to permit adherence prior to treatment and viral infection. Cells were next infected with HIV-1 NL4–3 or BaL strains (MOI 0.5 and 0.3, respectively) and incubated at 37 °C. Compounds (10 μM) and the reference compounds DS (12.5 μM, 100 folds its IC_50_, Sigma-Aldrich) and NEV (7.5 μM, 100 folds its IC_50_, Sigma-Aldrich) were added at different hours (0, 1, 2, 3, 4, 6, 8, and 24 h) post infection. Compound concentrations were chosen according to previous published data,^55^ showing that the ideal compound concentration in a TOA experiment should be 10- to 100-fold their IC_50_ values but should also be nontoxic to the cells. Following these criteria, we used the highest possible concentrations with the lowest cytotoxicity. After 25 h, cells were washed with PBS 1× and HIV-1 production was assessed following the LTR-luciferase signal using the britelite plus Reporter Gene Assay System (PerkinElmer, Waltman, MA, USA) according to the manufacturer’s protocol.

## Supporting Information Available

List of oligonucleotides, additional FRET and CD analysis, additional cytotoxicity and antiviral data, and compounds’ characterization (PDF)
