# Supplementary material for: Naphthalene Diimide–Tetraazacycloalkane Conjugates Are G-Quadruplex-Based HIV-1 Inhibitors with a Dual Mode of Action
Source: ACS Infect Dis. 2024 Jan 4;10(2):489–99. doi: 10.1021/acsinfecdis.3c00453 (PMC10862543; doi:10.1021/acsinfecdis.3c00453)
Supplement: Supplementary file 1 — id3c00453_si_001.pdf [file id3c00453_si_001.pdf]

Supporting Information

Naphthalene diimide-tetraazacycloalkane conjugates are G-quadruplex-based HIV-1 inhibitors with a dual mode of action

Matteo Nadai[a♣], Filippo Doria[b♣], Ilaria Frasson[a], Rosalba Perrone[c], Valentina Pirota[b], Greta Bergamaschi[d], Mauro Freccero[b*], Sara N. Richter[a*]

[a] *Department of Molecular Medicine, University of Padua, via Gabelli 63, 35121 Padua, Italy;*

[b] *Department of Chemistry, University of Pavia, V.le Taramelli 10, 27100 Pavia, Italy;*

[c] *Buck Institute for Research on Aging, Novato, CA 94945, USA*

[d] *National Research Council of Italy, Istituto di Scienze e Tecnologie Chimiche "Giulio Natta" (SCITEC-CNR), Via Mario Bianco 9, 20131 Milano, Italy.*

[e] *Microbiology and Virology Unit, Padua University Hospital, 35121 Padua, Italy.*

These authors contributed equally.

**corresponding authors. Email: mauro.freccero@unipv.it; sara.richter@unipd.it*

Table of content

Tables S1-S2. List of oligonucleotides used in this study S3

Figure S1. FRET analysis of oligonucleotides in the absence/presence of **5**, **6**, **7** S4

Figure S2. FRET competition assay S4

Figure S3. CD spectra of Tel22 and F21T oligonucleotides S5

Figure S4. CD analysis of LTR-III G4 oligonucleotide in the absence/presence of **5**, **6**, **7** S6

Figure S5. CD analysis of LTR-IV G4 oligonucleotide in the absence/presence of **5**, **6**, **7** S7

Figure S6. CD analysis of Tel22 G4 oligonucleotide in the absence/presence of **5**, **6**, **7** S8

Figure S7. Evaluation of compounds **5**, **6**, **7** cytotoxicity S9

Figure S8. Evaluation of compounds **5**, **6**, **7** cytotoxicity and antiviral activity 24 h.p.i. S10

Figure S9. Evaluation of compounds **5**, **6**, **7** cytotoxicity and antiviral activity 48 h.p.i. S13

Figure S10. Evaluation of AMD3100 cytotoxicity and antiviral activity S16

Figure S11. Evaluation of parental NDI cytotoxicity and antiviral activity S17

Figure S12. Evaluation of $CuSO_4$ cytotoxicity and antiviral activity S18

Compounds' characterization S19-S28

HPLC, ^{1}H- and ^{13}C-NMR spectra of compound **5** S19-S22

HPLC, ESI-MS spectra of compound **6** S23-S25

HPLC, ESI-MS spectra of compound **7** S26-S28

Table S1. Oligonucleotides used in this study (FRET)

Name	Sequence 5' – 3'
**LTR-III**	FAM-GGGAGGCGTGGCCTGGGCGGGACTGGGG-TAMRA
**LTR-IV**	FAM-TGGGCGGGACTGGGGAGTGGT-TAMRA
**F21T**	FAM-GGGTTAGGGTTAGGGTTAGGG-TAMRA
**dsDNA**	FAM-CAATCGGATCGAATTCGATCCGATTG-TAMRA

Table S2. Oligonucleotides used in this study (CD)

Name	Sequence 5' – 3'
**LTR-III**	GGGAGGCGTGGCCTGGGCGGGACTGGGG
**LTR-IV**	TGGGCGGGACTGGGGAGTGGT
**Tel22**	AGGGTTAGGGTTAGGGTTAGGG

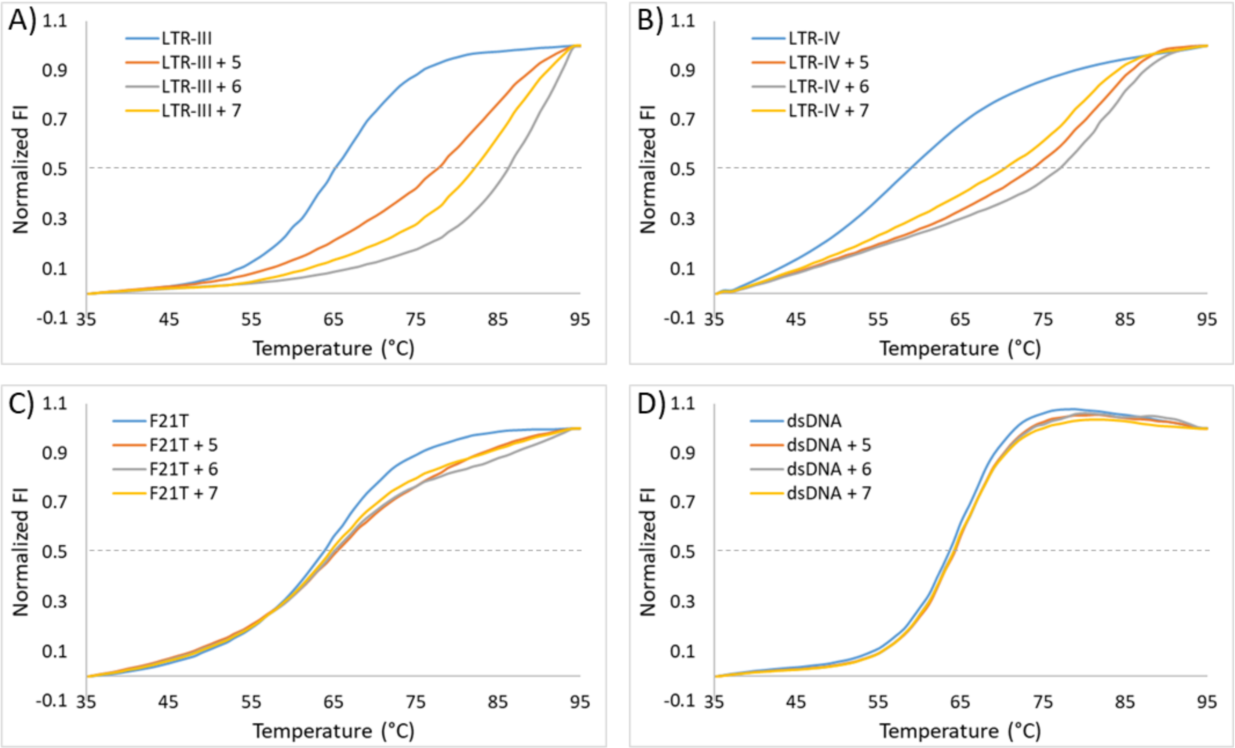

Figure S1. FRET melting spectra of oligonucleotide sequences (0.25 µM) in 100 mM KCl, alone or in presence of compounds (0.5 µM). A) LTR-III, B) LTR-IV, C) F21T, D) dsDNA.

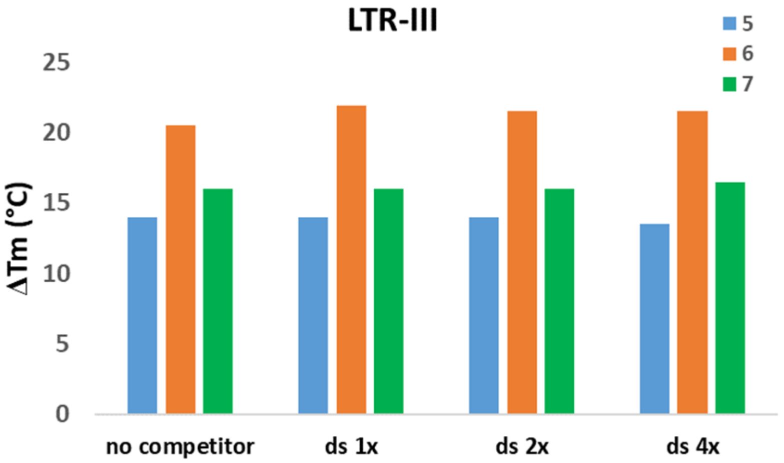

Figure S2. FRET competition assay. The fluorescent LTR-III oligonucleotide (0.25 µM) was incubated with compounds (0.5 µM) in the absence or presence of unlabeled ds26 (0.25 - 2 µM) and then subjected to FRET-melting. Graph represents ΔT_m values (°C).

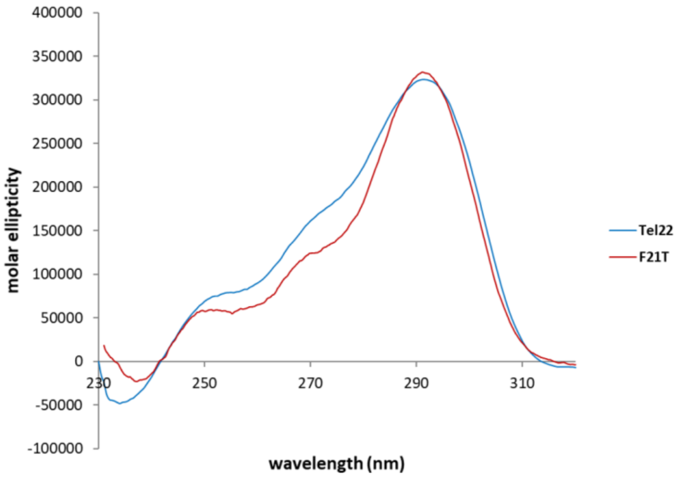

Figure S3. CD spectra of Tel22 and F21T oligonucleotides (4 μM) measured by circular dichroism in 100 mM KCl.

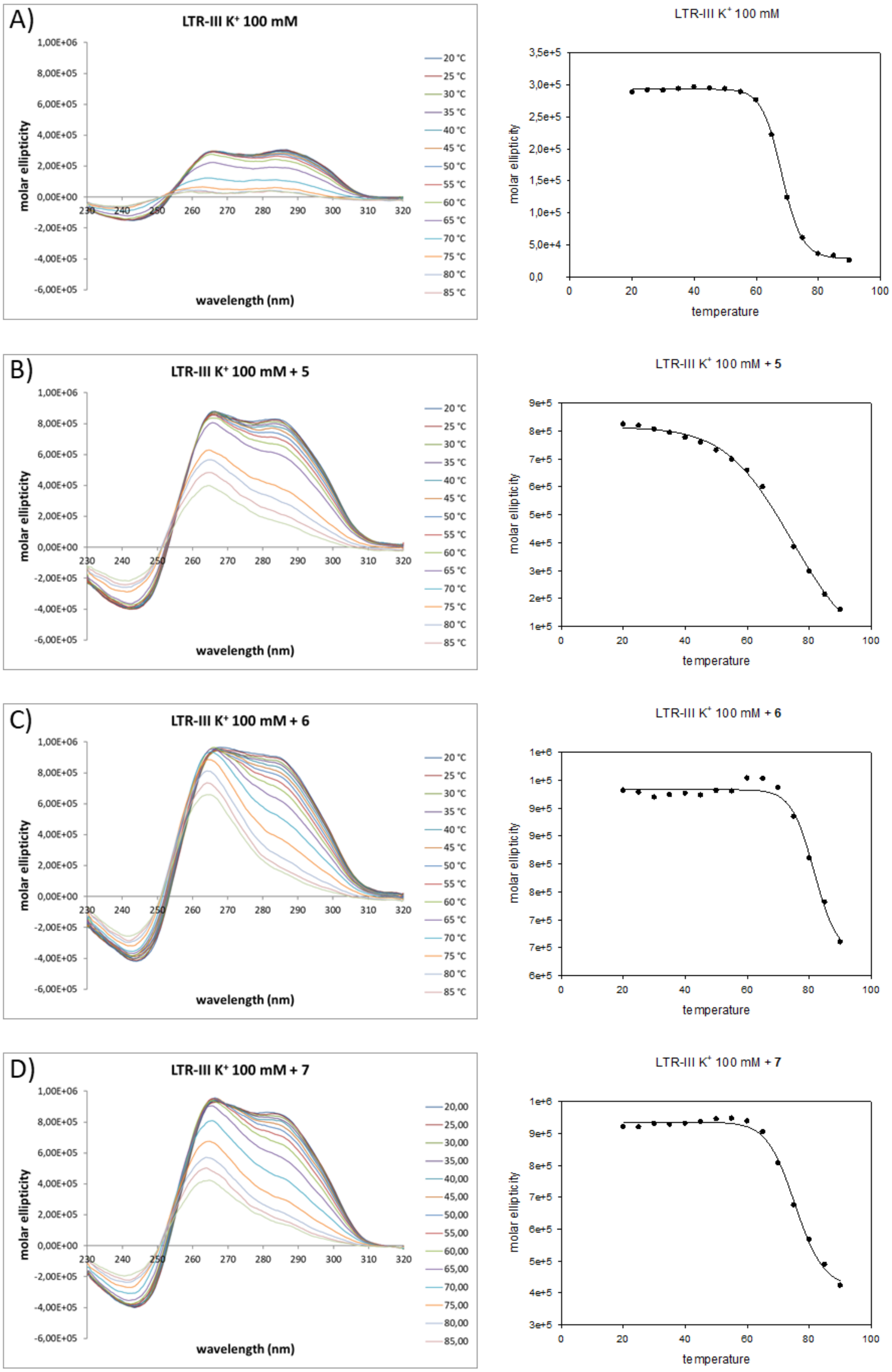

Figure S4. CD melting spectra (left) and fitting plots (right) of HIV-1 G4 folding sequence LTR-III (4 μM) measured by circular dichroism in 100 mM KCl, alone (A) or in the presence of compounds (8 μM) (B-D).

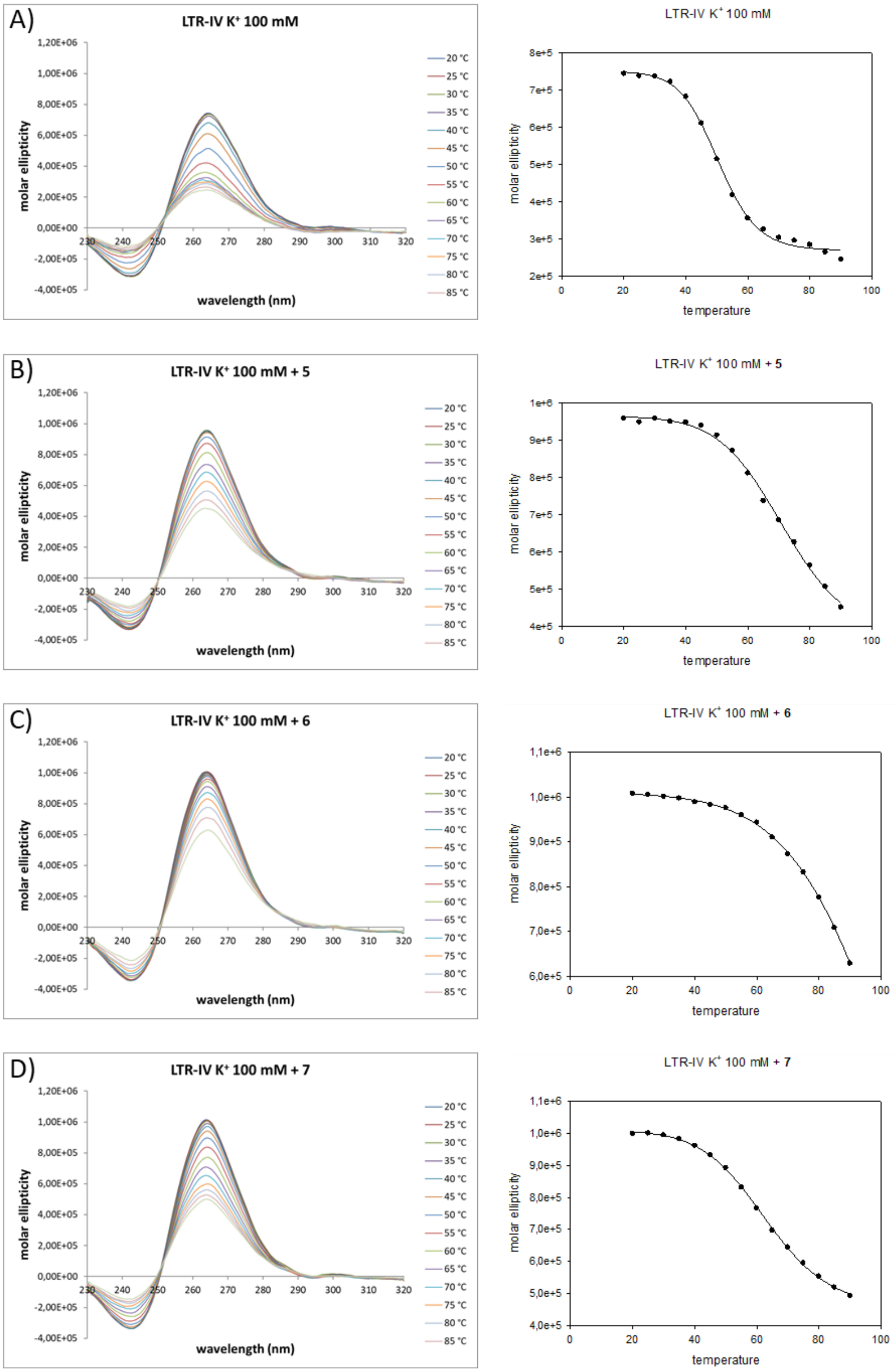

Figure S5. CD melting spectra (left) and fitting plots (right) of HIV-1 G4 folding sequence LTR-IV (4 μM) measured by circular dichroism in 100 mM KCl, alone (A) or in the presence of compounds (8 μM) (B-D).

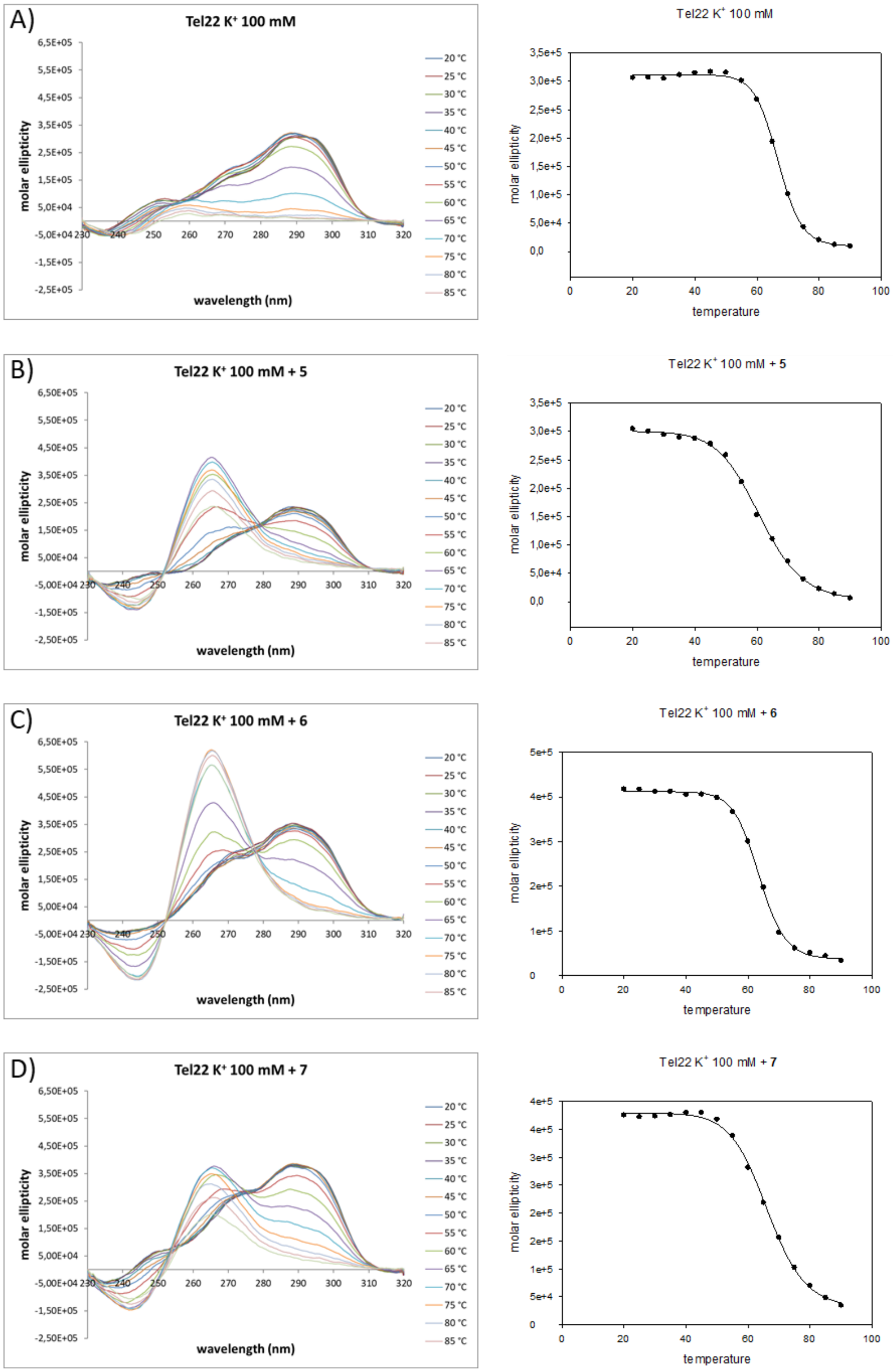

Figure S6. CD melting spectra (left) and fitting plots (right) of Tel22 sequence (4 µM) measured by circular dichroism in 100 mM KCl, alone (A) or in the presence of compounds (8 µM) (B-D).

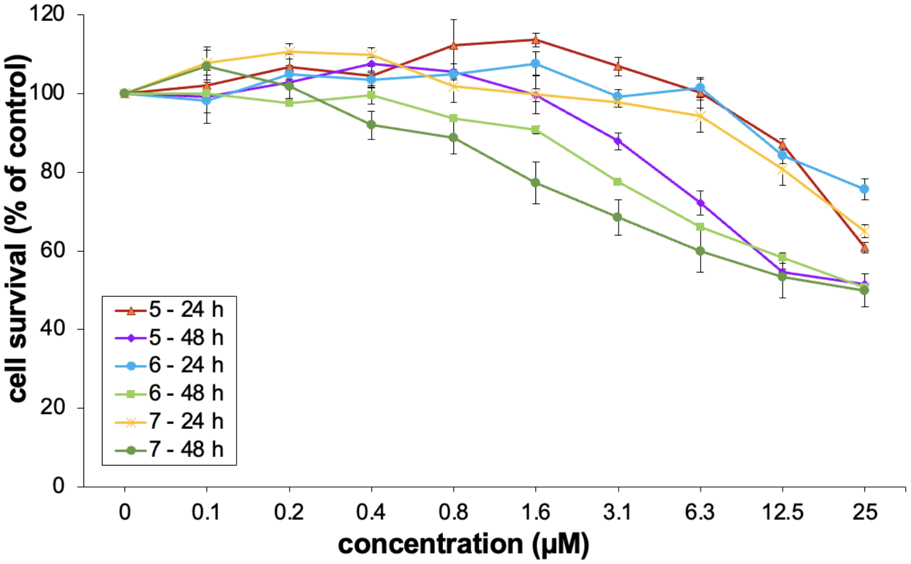

Figure S7. Evaluation of compounds' cytotoxicity. TZM-bl cells were treated with increasing concentrations of compounds **5-7**. After 24 and 48 h of treatment, cytotoxicity was assessed by MTT assay. Cytotoxicity of test compounds was compared with that of the mock-infected control.

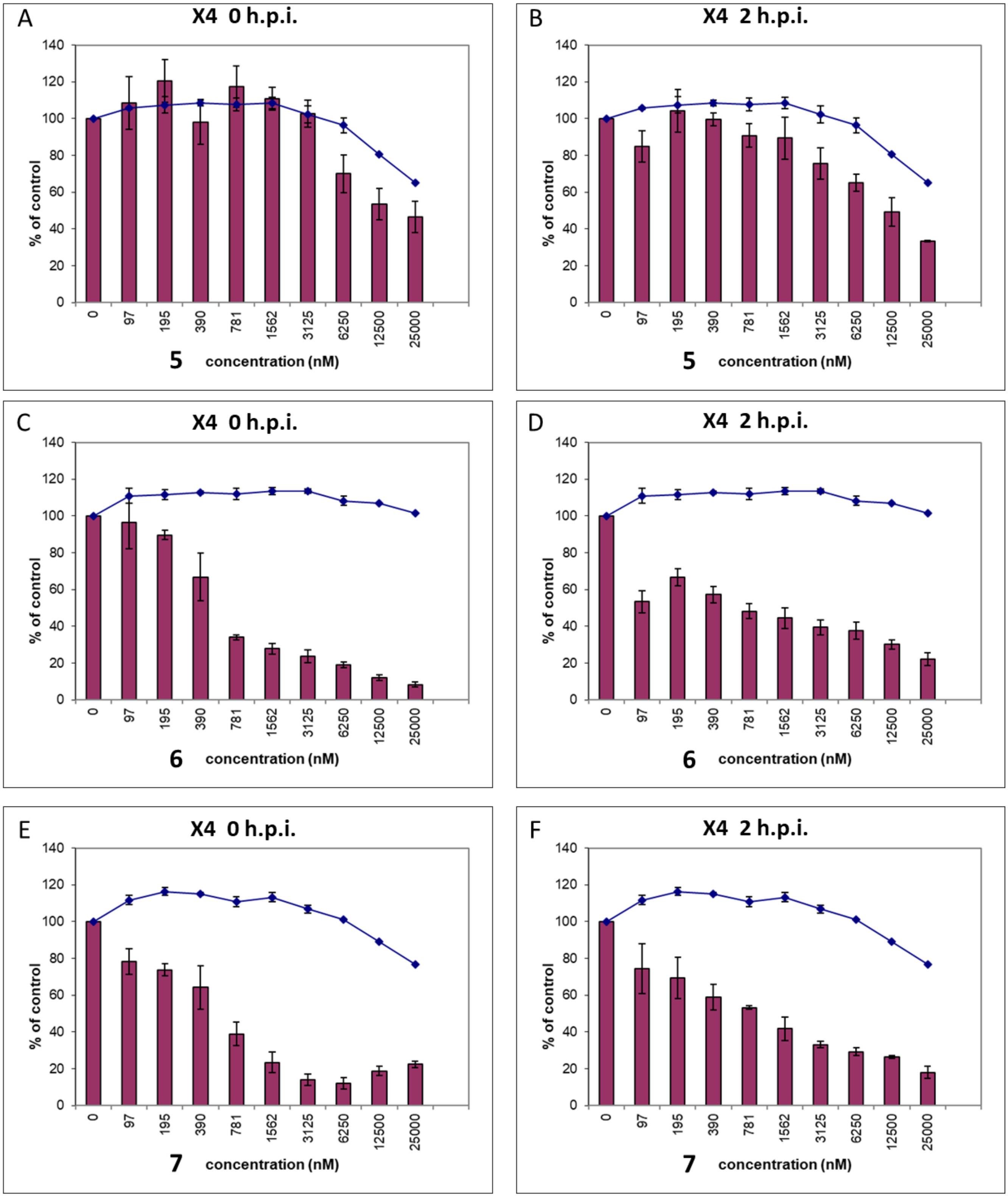
A
X4 0 h.p.i.
% of control
5 concentration (nM)
B
X4 2 h.p.i.
% of control
5 concentration (nM)
C
X4 0 h.p.i.
% of control
6 concentration (nM)
D
X4 2 h.p.i.
% of control
6 concentration (nM)
E
X4 0 h.p.i.
% of control
7 concentration (nM)
F
X4 2 h.p.i.
% of control
7 concentration (nM)
0 20 40 60 80 100 120 140
0 97 195 390 781 1562 3125 6250 12500 25000

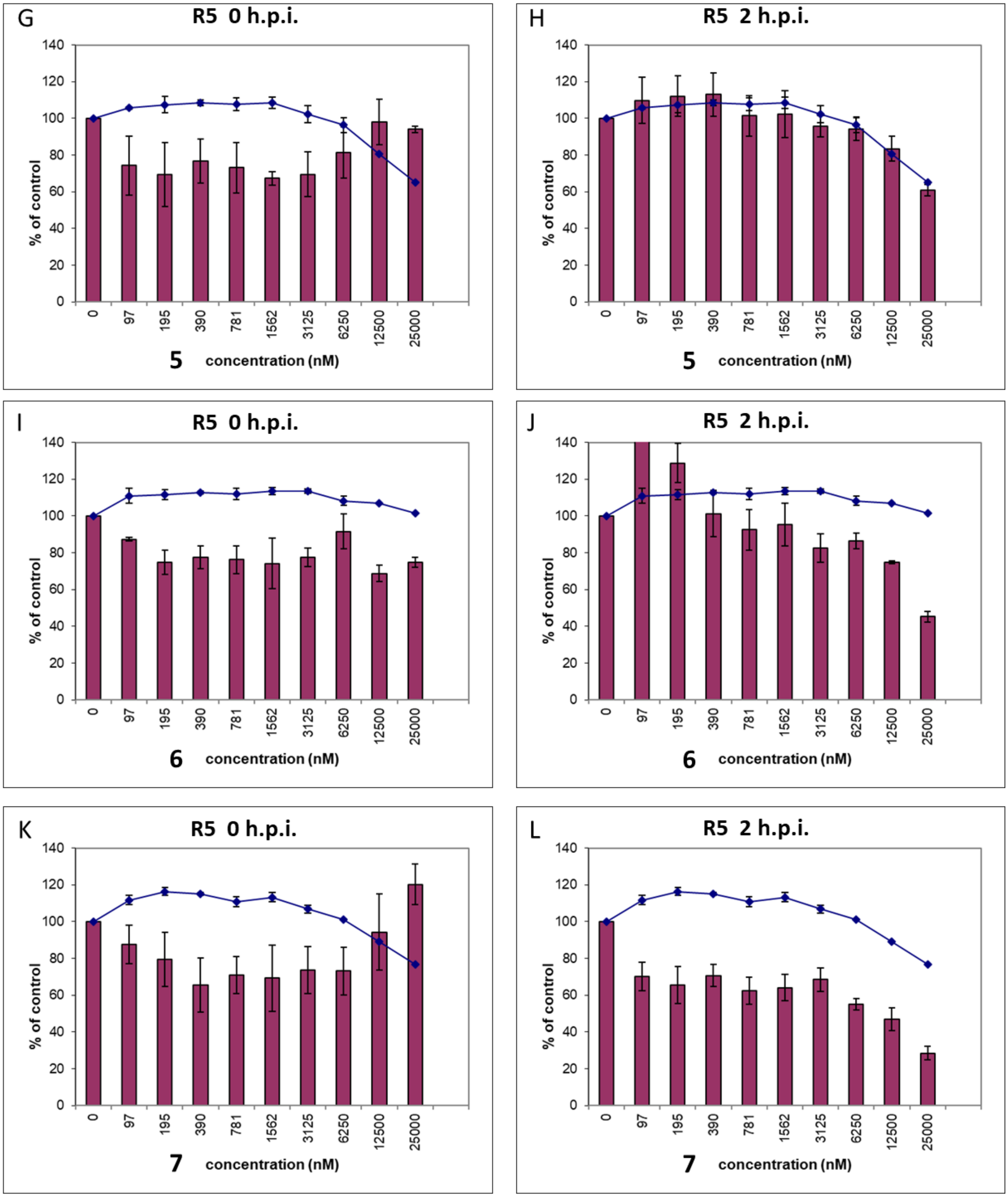

Figure S8. Evaluation of compounds **5**, **6**, **7** cytotoxicity and antiviral activity at 24 h.p.i. TZM-bl cells were infected with HIV-1 strain NL4-3 (X4) or strain BaL (R5) and treated with increasing concentrations of compound administered at the time of infection (panels A, C, E, G, I, K) or at 2 h.p.i.

(panels B, D, F, H, J, L). After 24 h of treatment, antiviral activity (purple bars) was assessed following the LTR-luciferase signal using the britelite plus Reporter Gene Assay System, while cytotoxicity (blue line) was assessed by MTT assay.

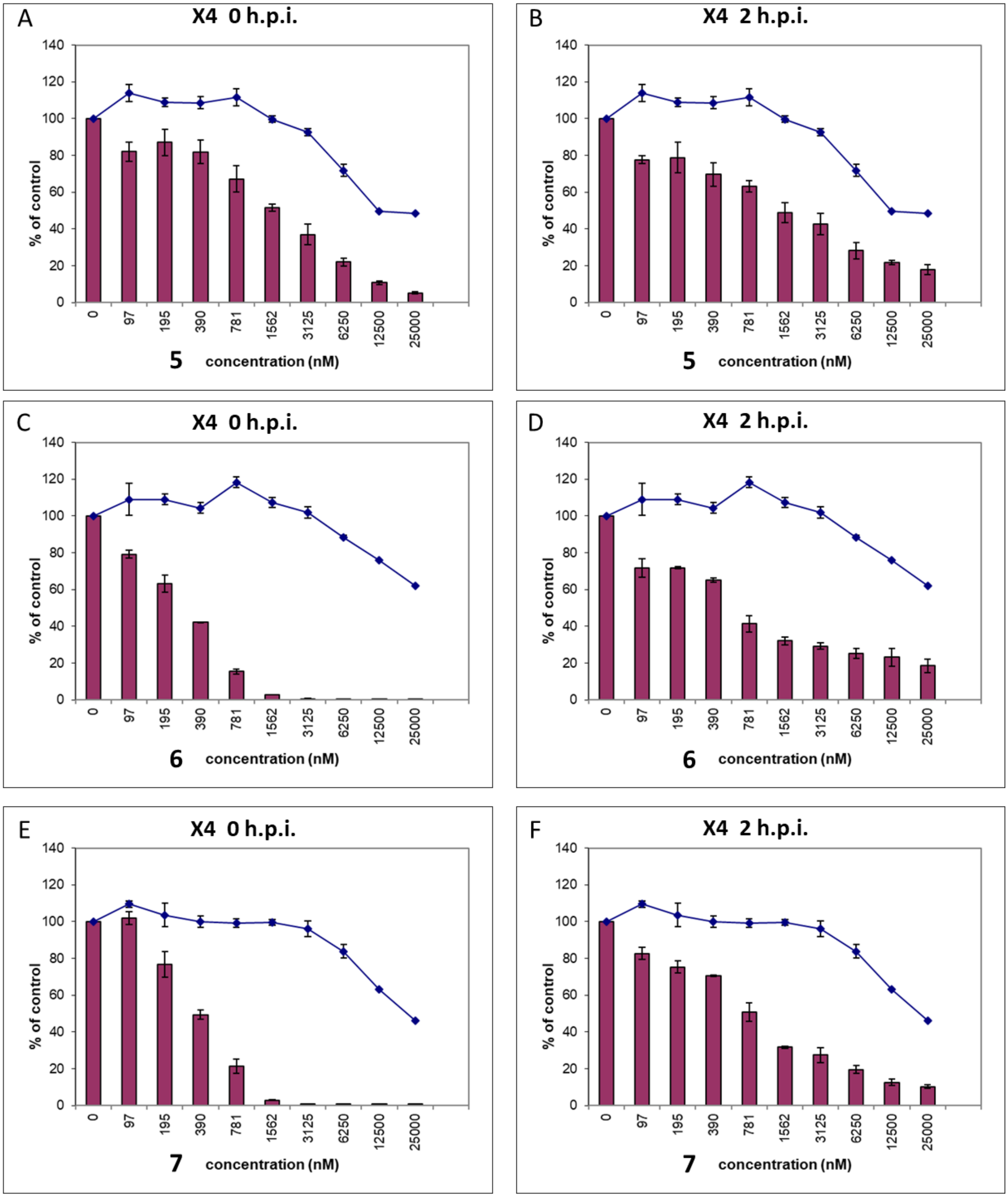
A
X4 0 h.p.i.
B
X4 2 h.p.i.
C
X4 0 h.p.i.
D
X4 2 h.p.i.
E
X4 0 h.p.i.
F
X4 2 h.p.i.
% of control
0
20
40
60
80
100
120
140
0
97
195
390
781
1562
3125
6250
12500
25000
5
6
7
concentration (nM)

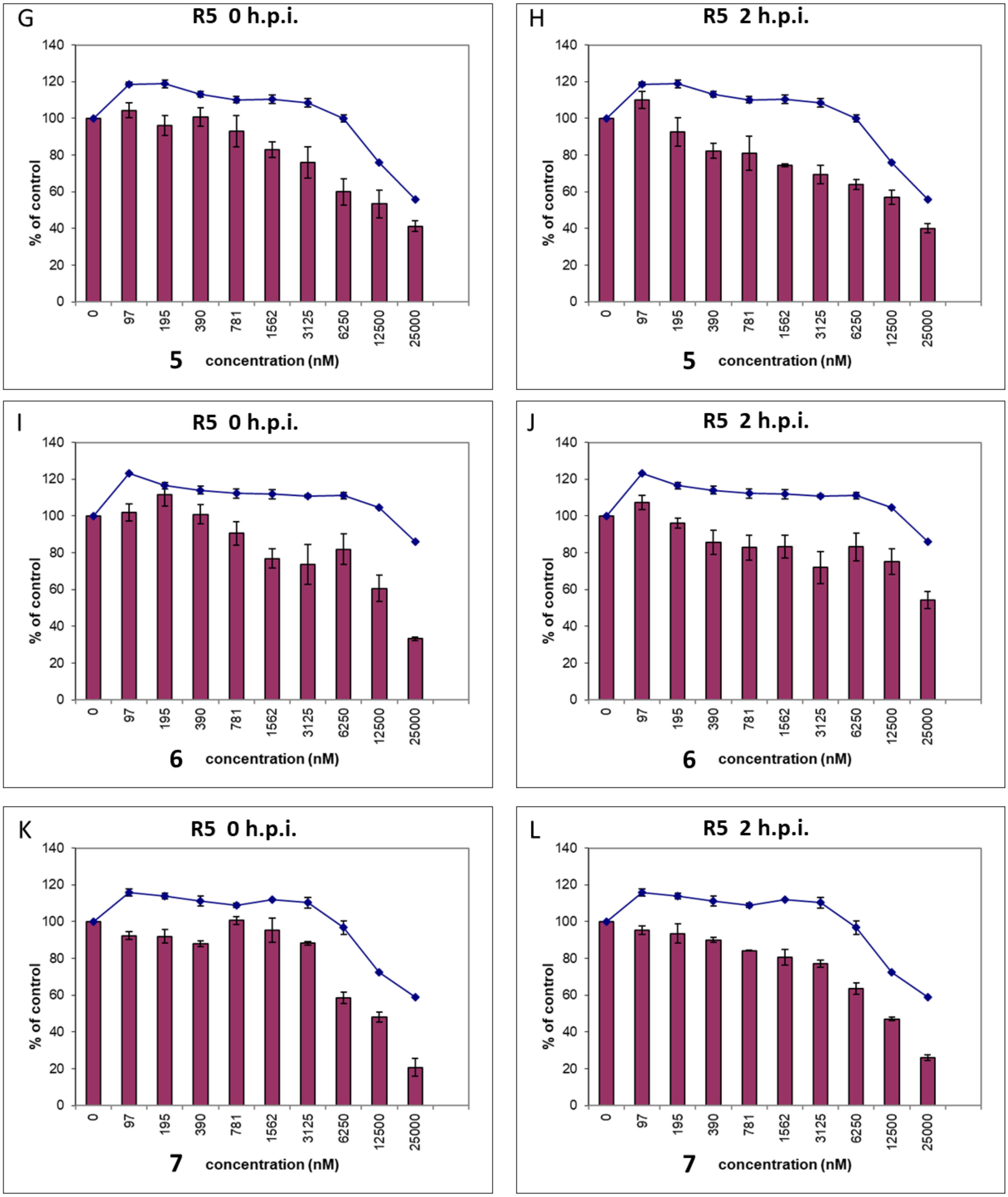

Figure S9. Evaluation of compounds **5**, **6**, **7** cytotoxicity and antiviral activity at 48 h.p.i. TZM-bl cells were infected with HIV-1 strain NL4-3 (X4) or strain BaL (R5) and treated with increasing concentrations of compound administered at the time of infection (panels A, C, E, G, I, K) or at 2 h.p.i.

(panels B, D, F, H, J, L). After 48 h of treatment, antiviral activity (purple bars) was assessed following the LTR-luciferase signal using the britelite plus Reporter Gene Assay System, while cytotoxicity (blue line) was assessed by MTT assay.

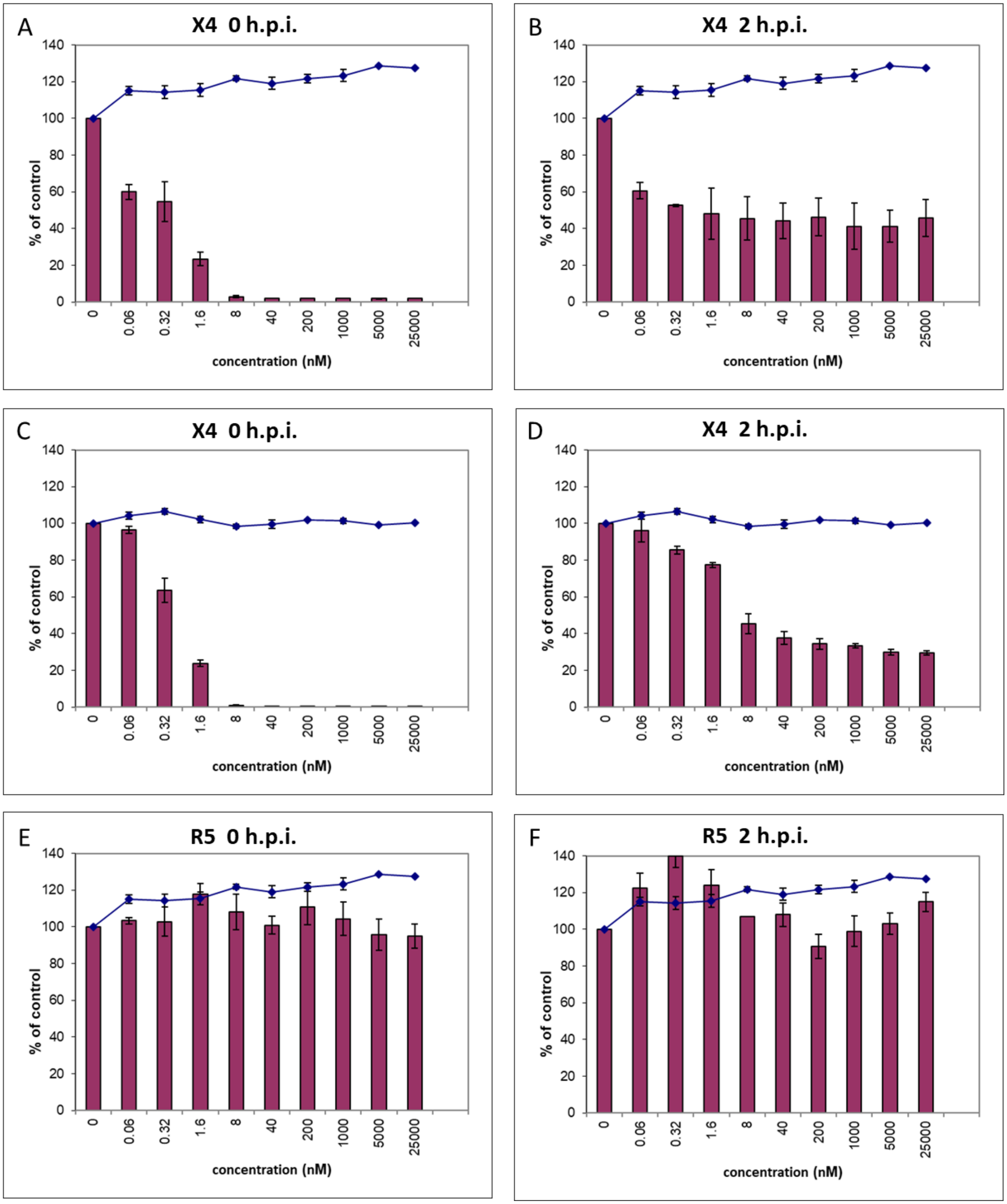

Figure S10. Evaluation of AMD3100 cytotoxicity and antiviral activity. TZM-bl cells were infected with HIV-1 strain NL4-3 (X4) or strain BaL (R5) and treated with increasing concentrations of compound administered at the time of infection (panels A, C, E) or 2 h.p.i. (panels B, D, F). After 24 h (panels A-

B, E-F) or 48 h (panels C-D) of treatment, antiviral activity (purple bars) was assessed following the LTR-luciferase signal using the britelite plus Reporter Gene Assay System, while cytotoxicity (blue line) was assessed by MTT assay.

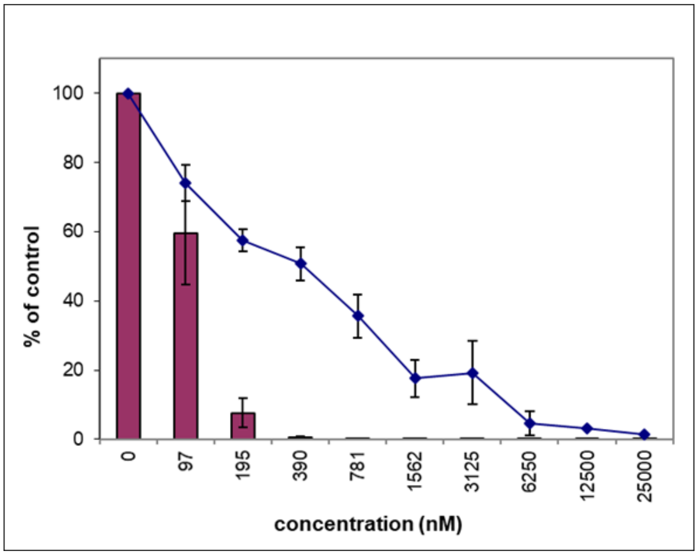

Figure S11. Evaluation of parental NDI cytotoxicity and antiviral activity. TZM-bl cells were infected with HIV-1 strain NL4-3 and treated with increasing concentrations of compound. After 24 h of treatment, antiviral activity (purple bars) was assessed following the LTR-luciferase signal using the britelite plus Reporter Gene Assay System, while cytotoxicity (blue line) was assessed by MTT assay.

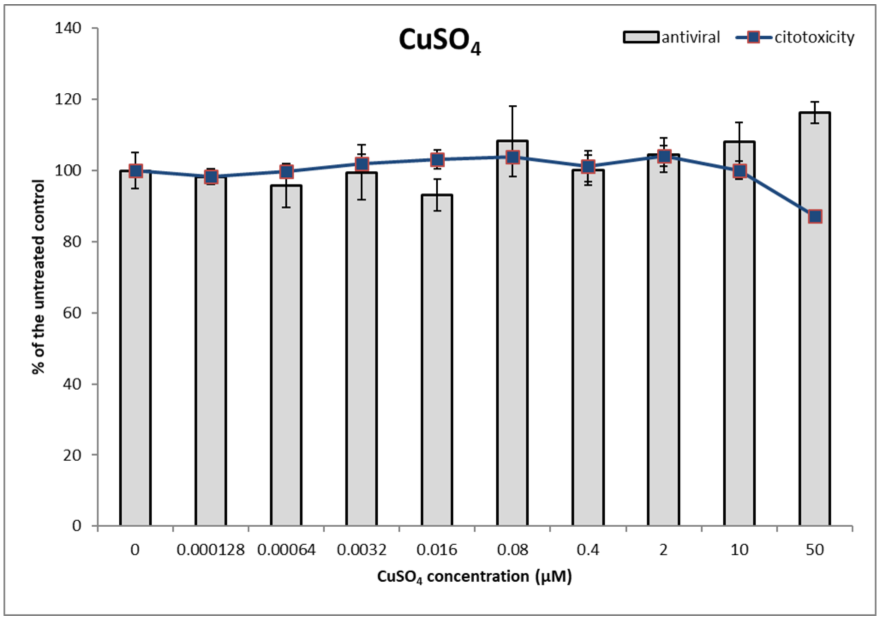

Figure S12. Evaluation of $CuSO_4$ cytotoxicity and antiviral activity. TZM-bl cells were infected with HIV-1 strain NL4-3 and $CuSO_4$ was added at indicated concentrations after infection. 48 h post-infection HIV-1 production was assessed following the LTR-luciferase signal using the britelite plus Reporter Gene Assay System, while cytotoxicity was assessed using MTT assay. The activity of $CuSO_4$ was compared with that of the negative control.

COMPOUNDS' CHARACTERIZATION

NDI-tetraazacycloalkane conjugate **5**

HPLC

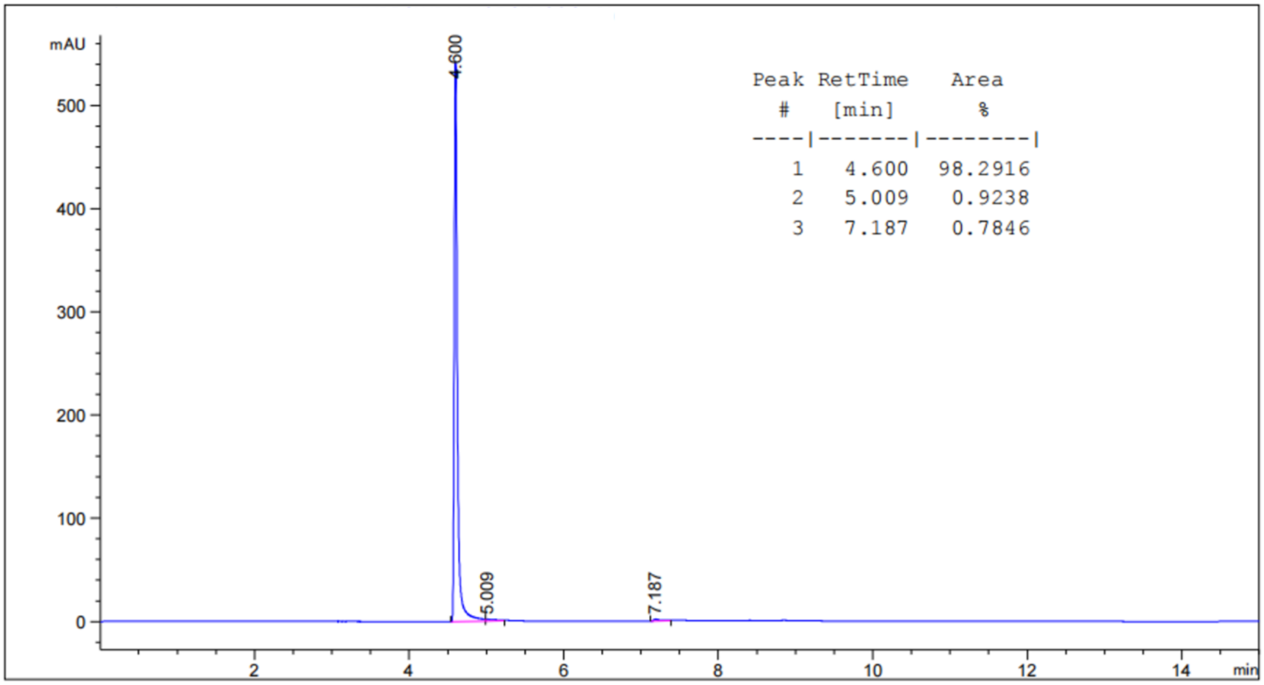

^{1}H-NMR (CD_3OD)

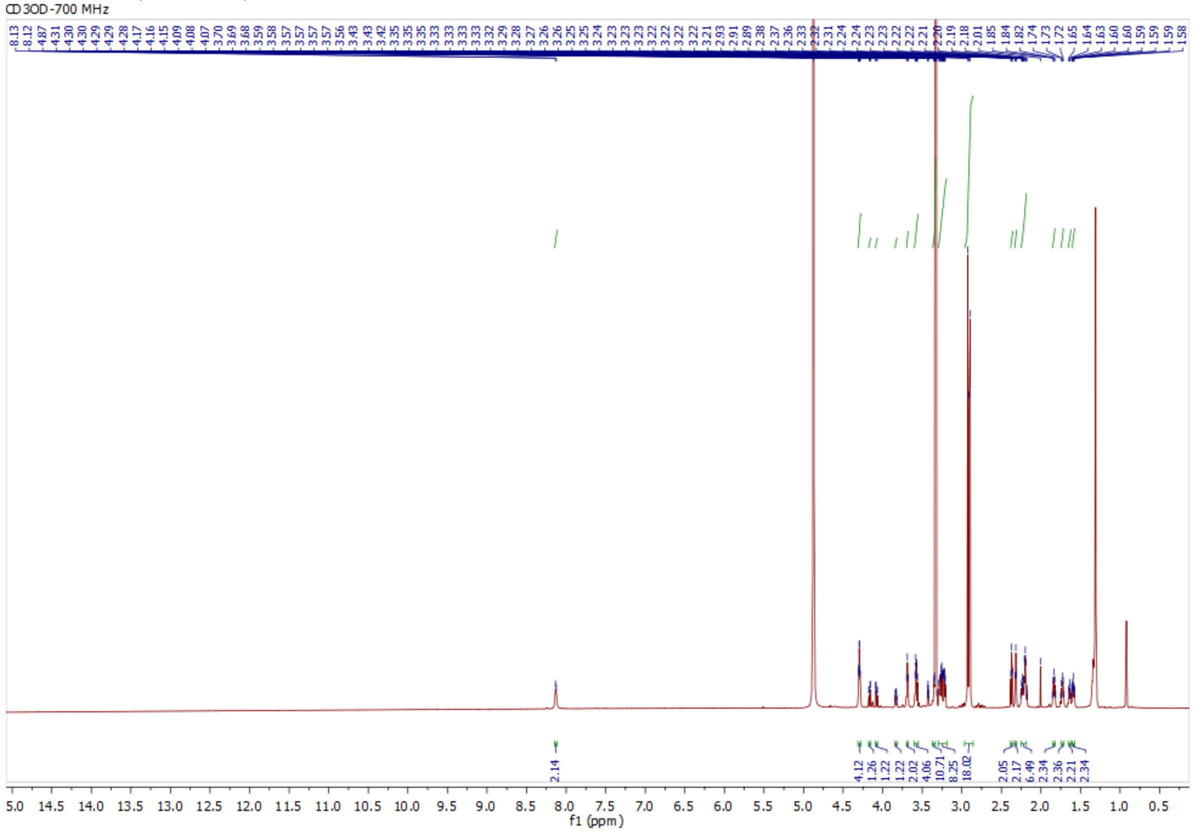

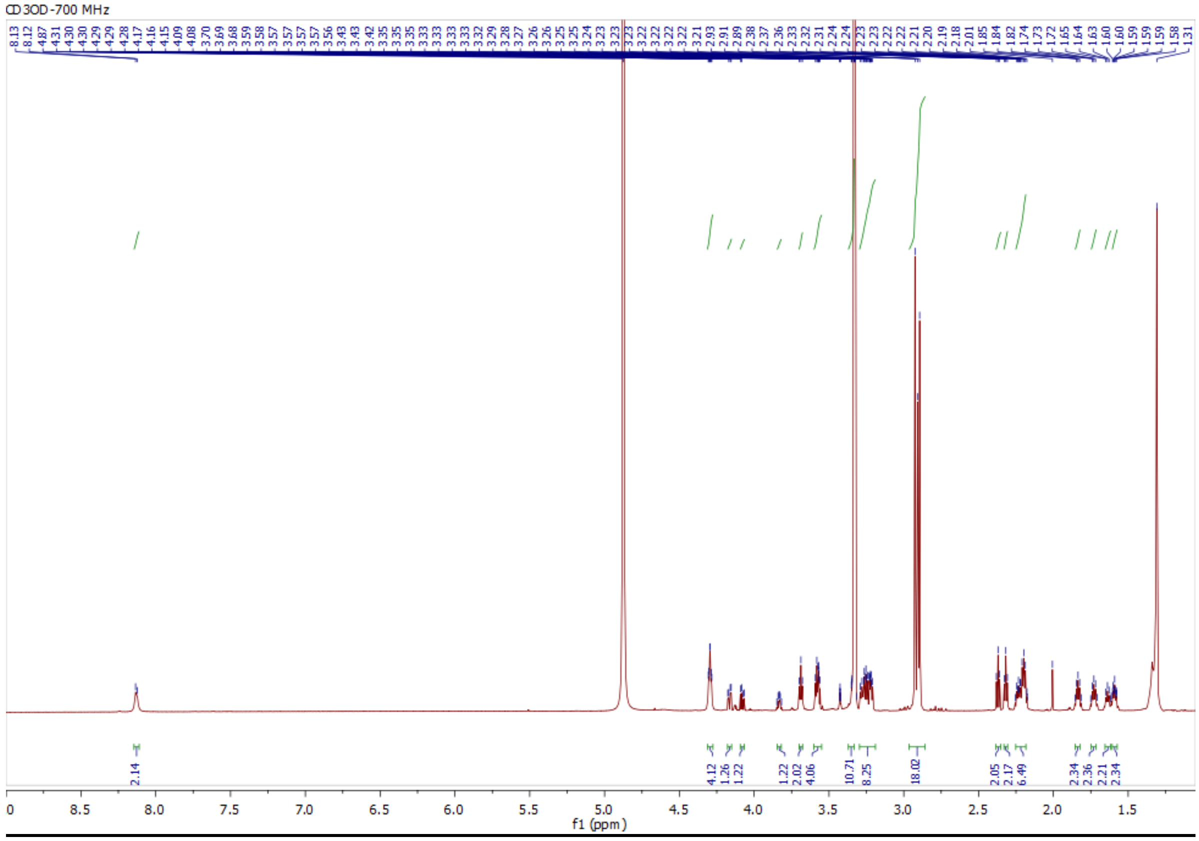

^{13}C-NMR (CD_3OD)

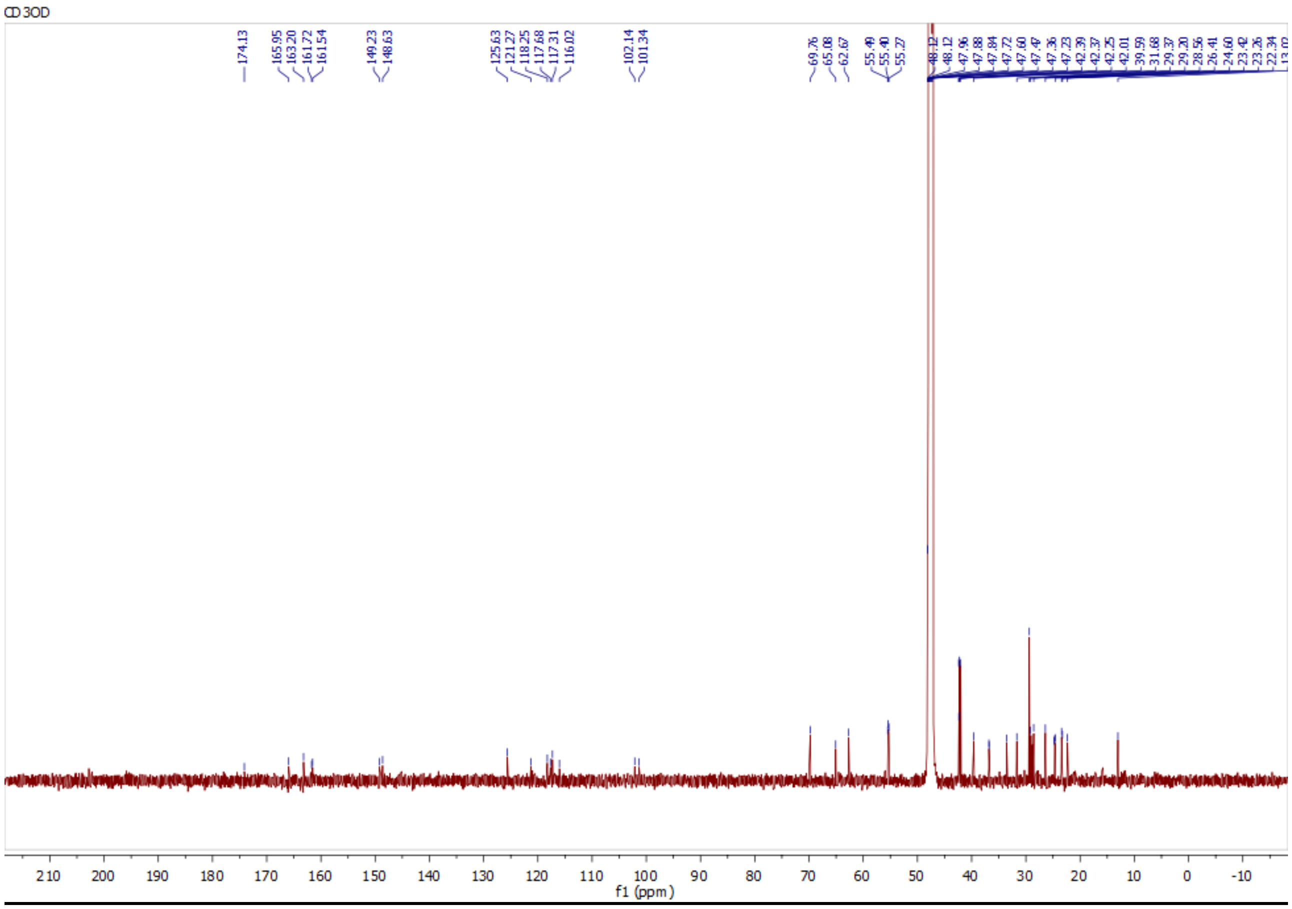

NDI-MOC conjugate incorporating Cu^{2+} **6**

HPLC

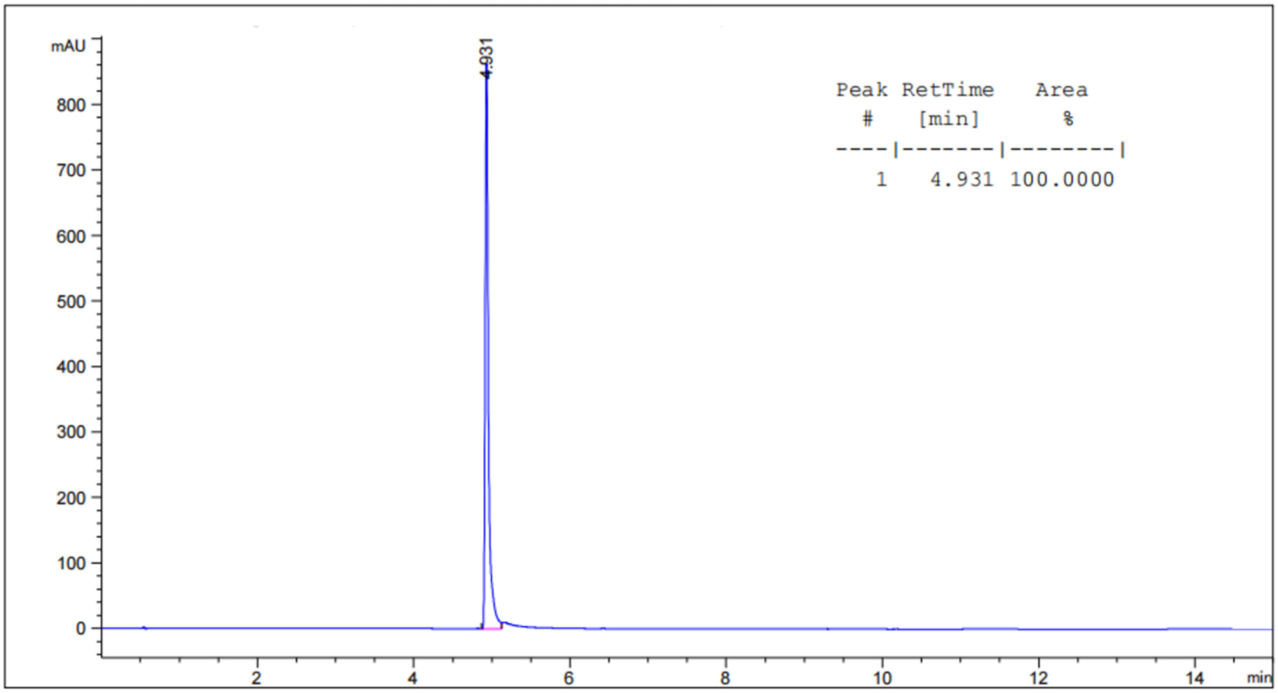

ESI-MS

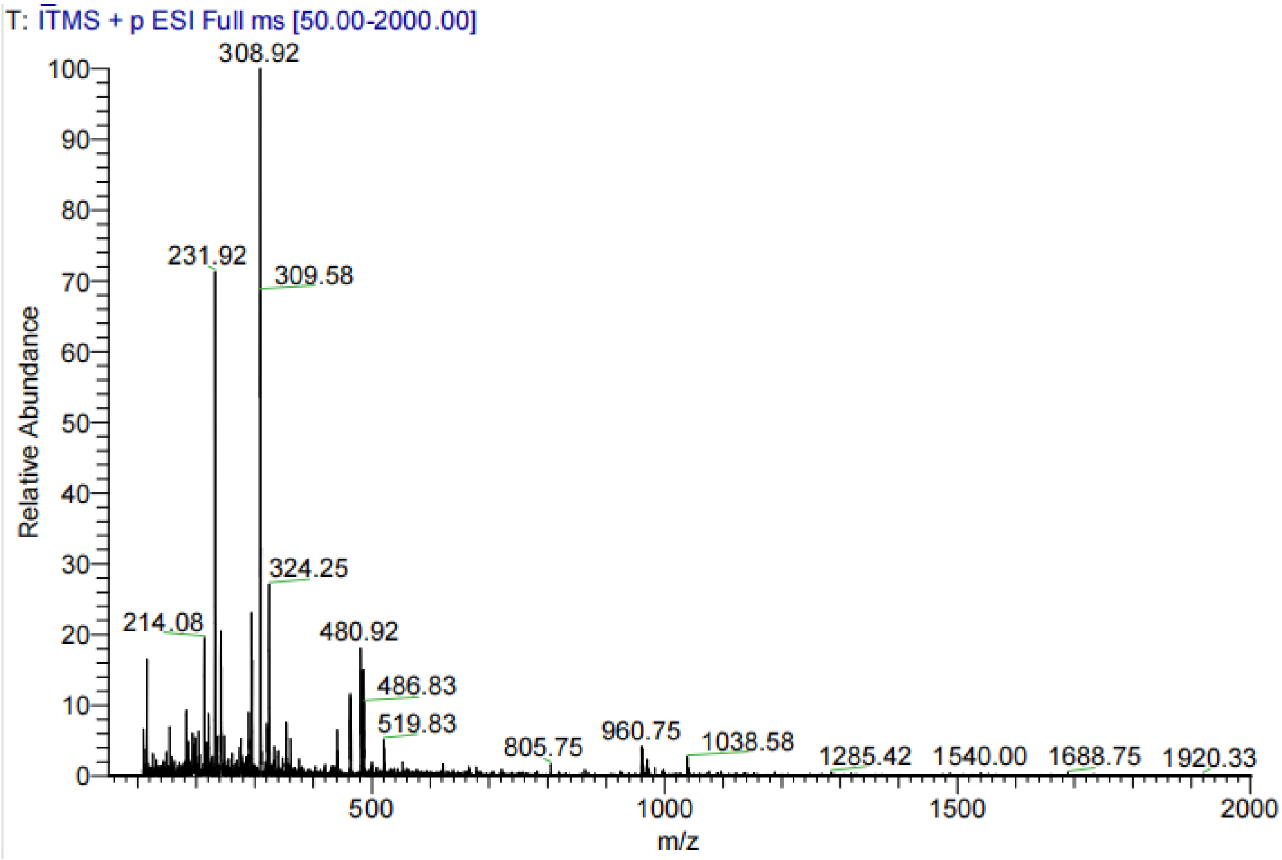
T: ITMS + p ESI Full ms [50.00-2000.00]
308.92
231.92
309.58
324.25
214.08
480.92
486.83
519.83
805.75
960.75
1038.58
1285.42
1540.00
1688.75
1920.33
Relative Abundance
m/z
0
10
20
30
40
50
60
70
80
90
100
500
1000
1500
2000

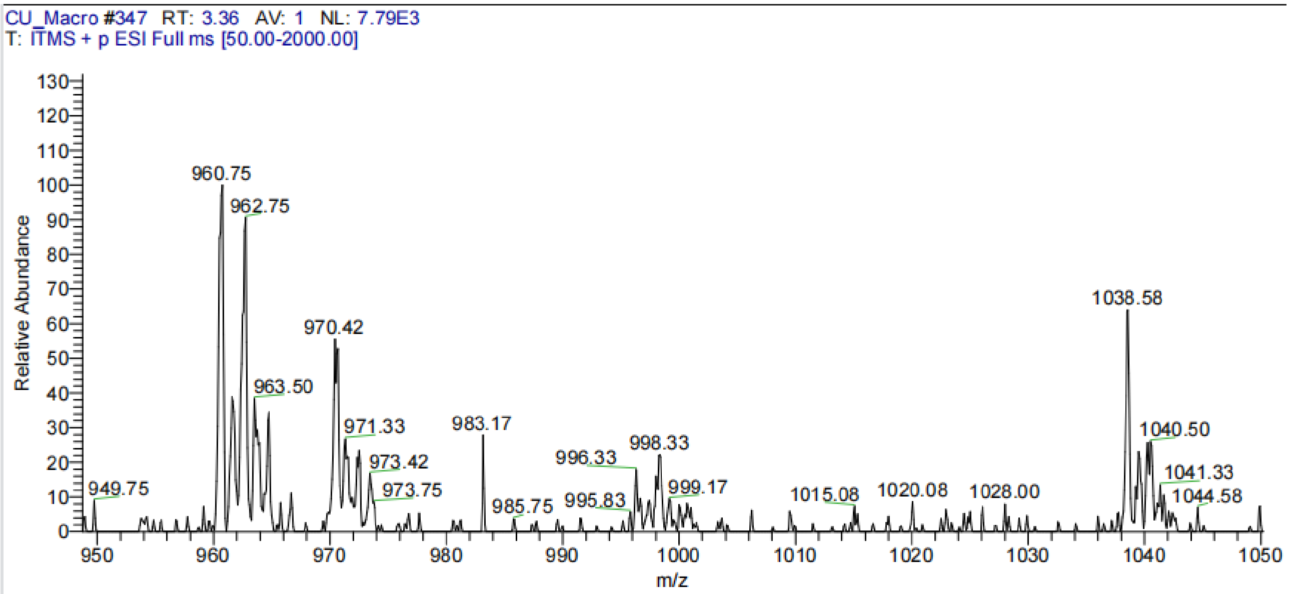
CU_Macro #347 RT: 3.36 AV: 1 NL: 7.79E3
T: ITMS + p ESI Full ms [50.00-2000.00]
Relative Abundance
m/z
960.75
962.75
963.50
970.42
971.33
973.42
973.75
983.17
985.75
995.83
996.33
998.33
999.17
949.75
1015.08
1020.08
1028.00
1038.58
1040.50
1041.33
1044.58

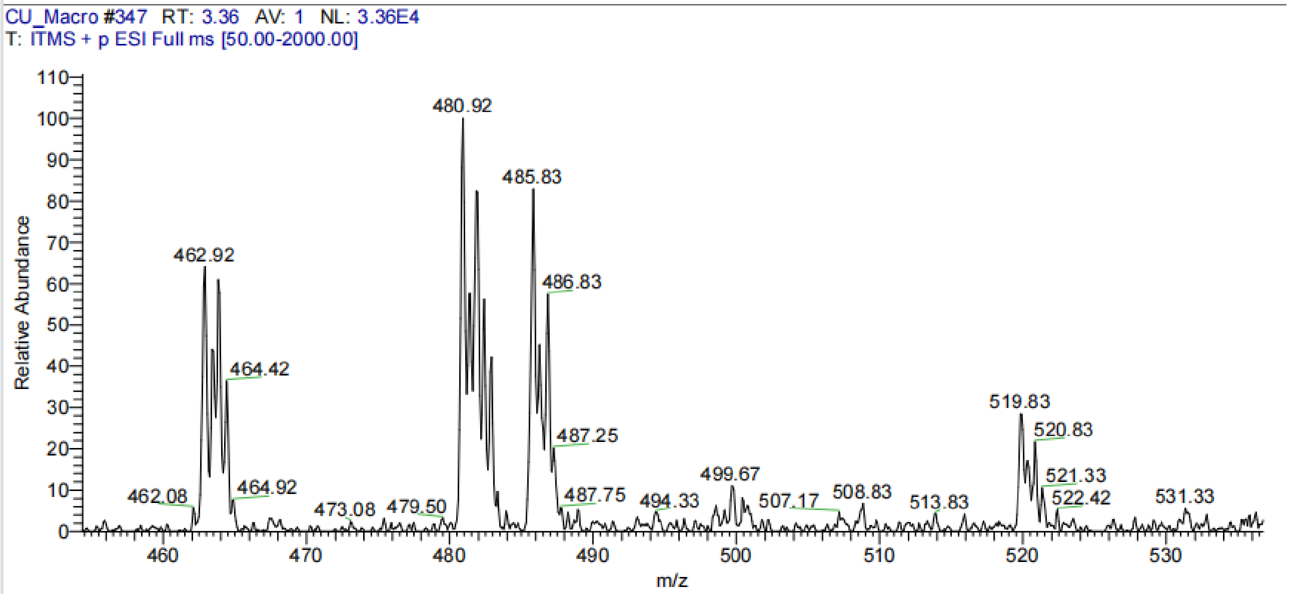
CU_Macro #347 RT: 3.36 AV: 1 NL: 3.36E4
T: ITMS + p ESI Full ms [50.00-2000.00]
Relative Abundance
m/z
480.92
485.83
462.92
486.83
464.42
487.25
462.08
464.92
473.08
479.50
487.75
494.33
499.67
507.17
508.83
513.83
519.83
520.83
521.33
522.42
531.33

NDI-MOC conjugate incorporating Zn^{2+} **7**

HPLC

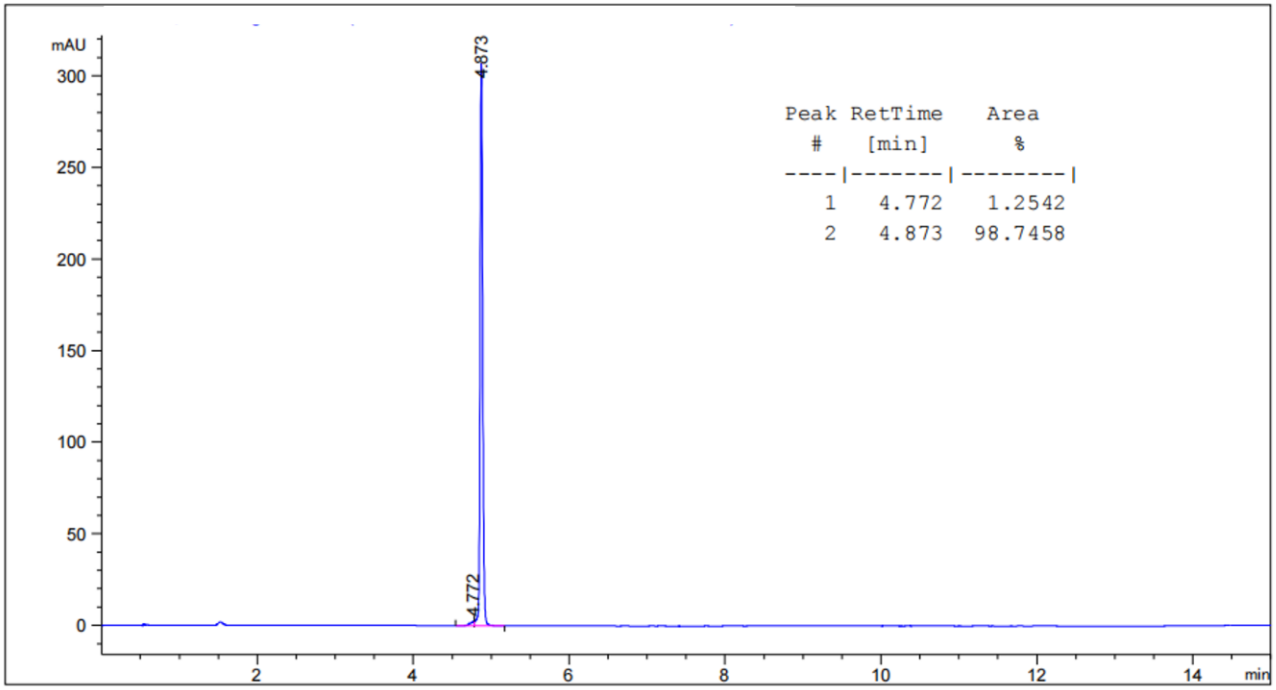

ESI-MS

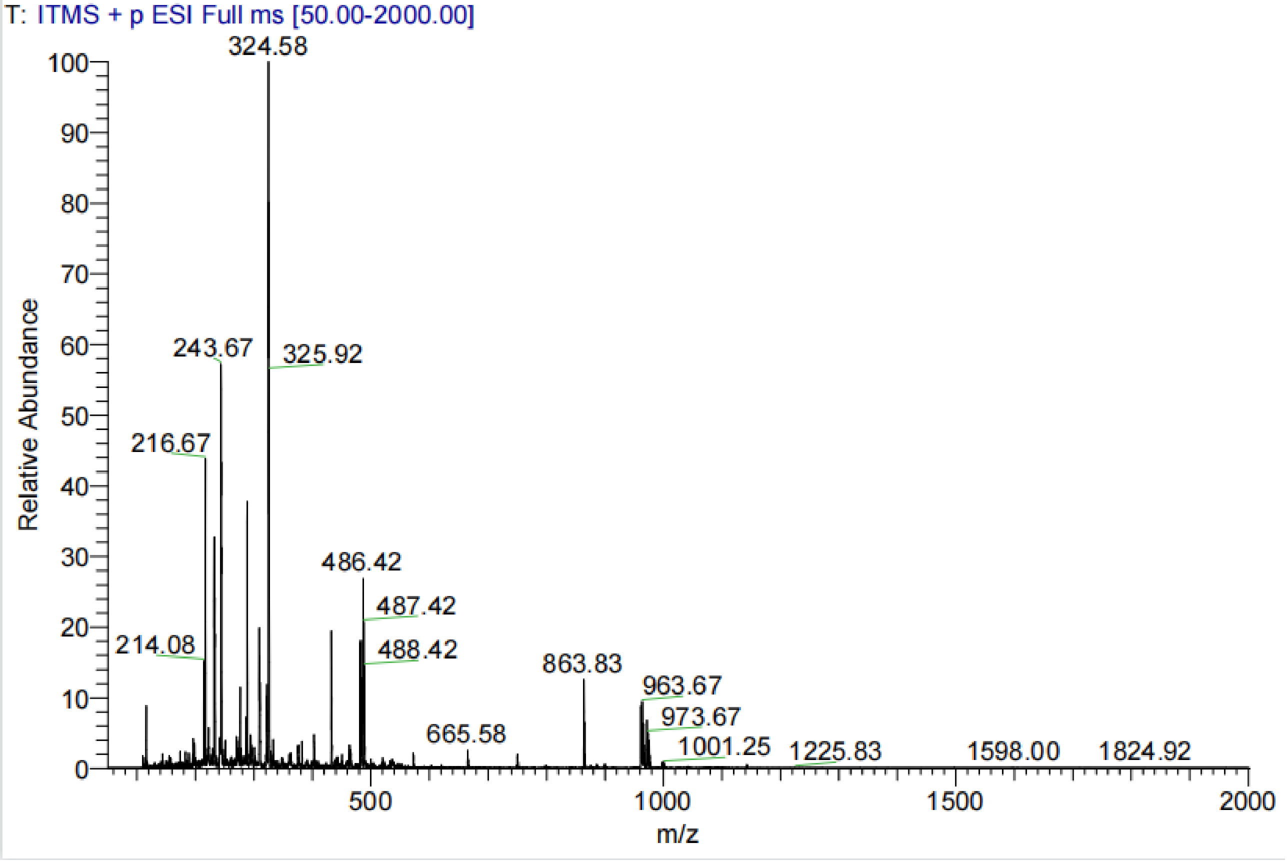

T: ITMS + p ESI Full ms [50.00-2000.00]
324.58
243.67
325.92
216.67
486.42
487.42
214.08
488.42
863.83
963.67
973.67
665.58
1001.25
1225.83
1598.00
1824.92
Relative Abundance
m/z

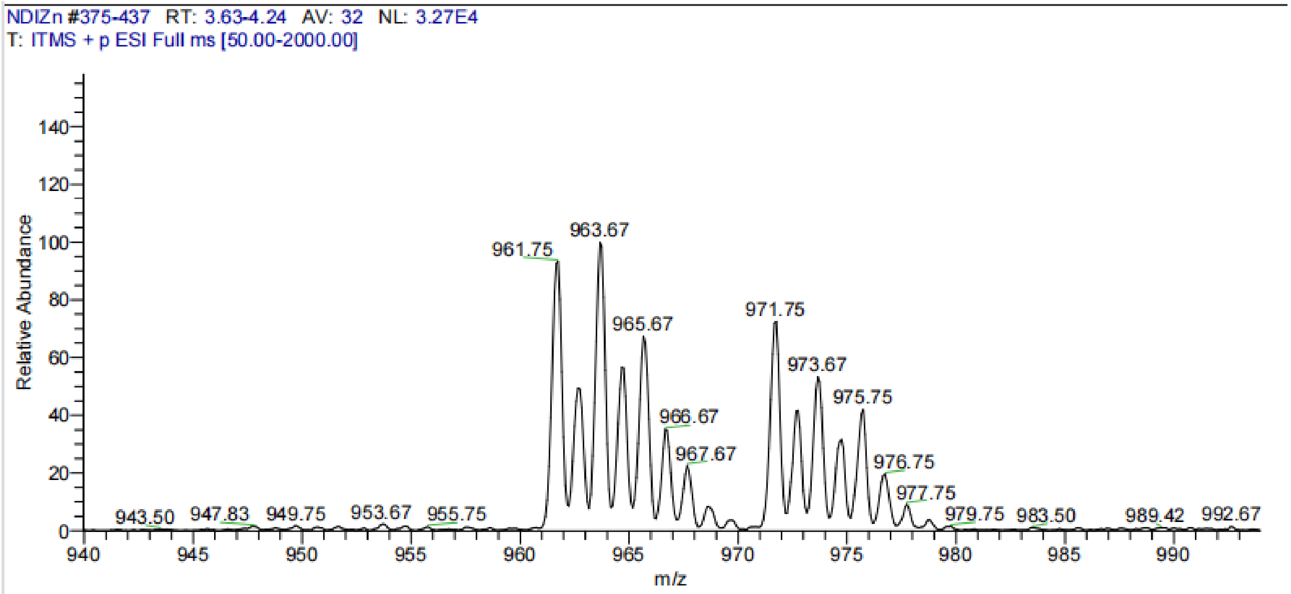
NDIZn #375-437 RT: 3.63-4.24 AV: 32 NL: 3.27E4
T: ITMS + p ESI Full ms [50.00-2000.00]
Relative Abundance
m/z
961.75
963.67
965.67
966.67
967.67
971.75
973.67
975.75
976.75
977.75
943.50
947.83
949.75
953.67
955.75
979.75
983.50
989.42
992.67

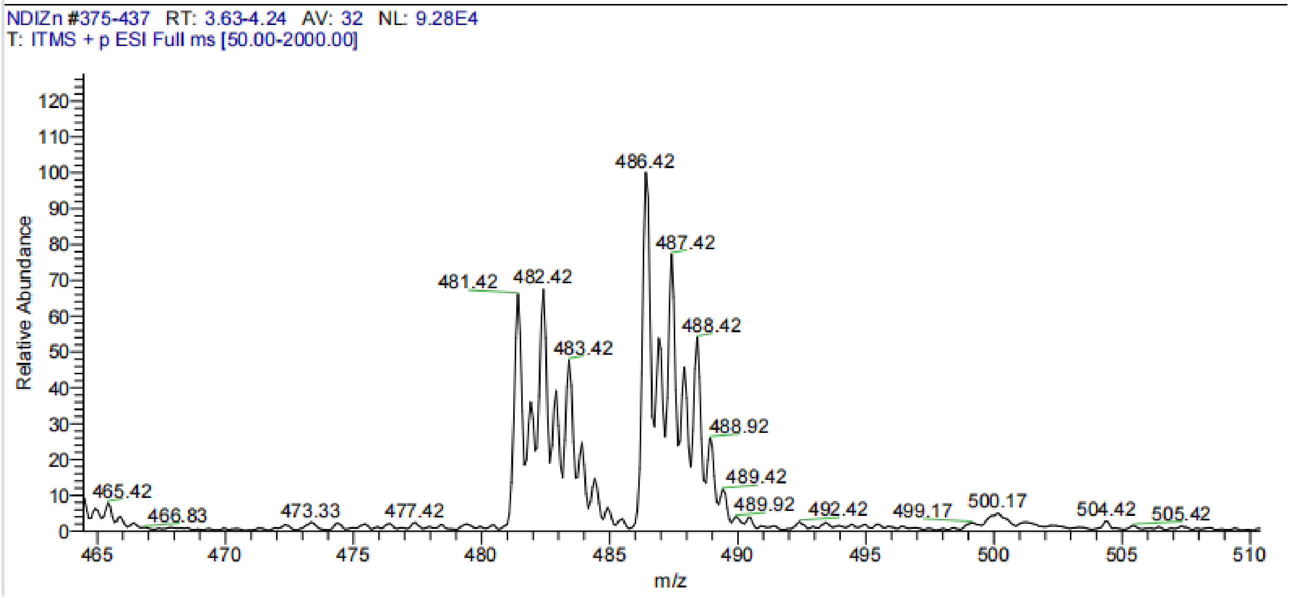
NDIZn #375-437 RT: 3.63-4.24 AV: 32 NL: 9.28E4
T: ITMS + p ESI Full ms [50.00-2000.00]
Relative Abundance
m/z
486.42
487.42
481.42
482.42
483.42
488.42
488.92
489.42
489.92
465.42
466.83
473.33
477.42
492.42
499.17
500.17
504.42
505.42
